# Microbiome and metabolome dynamics in phloem and rhizosphere of *Pinus tabuliformis* against *Dendroctonus valens* infestation

**DOI:** 10.3389/fmicb.2026.1754801

**Published:** 2026-03-04

**Authors:** Yiru Han, Hui Huang, Zhiwei Zhang, Xinyu Li, Tao Li, Shixiang Zong

**Affiliations:** 1 Beijing Key Laboratory for Forest Pest Control, Beijing Forestry University, Beijing, China; 2 College of Forestry, Shanxi Agricultural University, Taiyuan, China; 3 Center for Biological Disaster Prevention and Control, National Forestry and Grassland Administration, Shenyang, China; 4 State Key Laboratory to Efficient Production of Forest Resources, Beijing Forestry University, Beijing, China

**Keywords:** *Dendroctonus valens*, macromolecular defense, metabolome, microbiome, phloem, *Pinus tabuliformis*, rhizosphere soil

## Abstract

Microbial communities play essential roles in mediating plant defenses against insect pests. However, how host-associated microbiota and metabolites jointly respond to bark beetle infestation remains largely unexplored. Here, we integrated microbiome and metabolome profiling to elucidate how *Pinus tabuliformis* regulates its phloem and rhizosphere responses under varying levels of *Dendroctonus valens* infestation. Both bacterial and fungal diversity, as well as the relative abundance of dominant taxa such as *Erwinia* and *Pseudoxanthomonas*, shifted significantly with infestation intensity. Concurrently, key plant defense metabolites—including terpenoids, jasmonates, and polyphenols—were markedly elevated. Pathway enrichment analysis indicated that the phloem was characterized by enhanced phenylpropanoid and flavonoid biosynthesis, whereas the rhizosphere soil accumulated terpenoids and polyketides, implicating both compartments in resistance modulation. In the phloem, differential bacterial and fungal taxa displayed distinct positive and negative correlations with phenylpropanoid intermediates and downstream derivatives, while in the rhizosphere, bacteria from Bacillota and fungi such as *Candida* and *Ogataea* were strongly linked to diterpenoids, sesquiterpenoids, flavonoids, and indole derivatives. These findings demonstrate that *P. tabuliformis* mounts a compartment-specific, microbiome-associated metabolic response to *D. valens* infestation, providing new insights into the ecological roles of symbiotic microbiota in plant defense and offering a mechanistic foundation for microbe-based pest management strategies.

## 1 Introduction

Microbial biocontrol has become a cornerstone of sustainable pest management, offering an effective alternative to conventional chemical pesticides (Naranjo et al., 2015). Insect infestation not only causes direct physiological injury to plants but also elicits complex biochemical and macromolecular defense responses (Jing et al., 2024). These responses extend beyond the damaged tissues, influencing plant health and resilience by reshaping the composition and metabolic activity of rhizosphere microbial communities (Harmsen et al., 2024). In particular, insect feeding alters the functional potential of rhizosphere microbiota, thereby affecting plant nutrient acquisition and the biosynthesis of defensive macromolecules such as terpenoids, flavonoids, and phenolics (Abbas et al., 2024). Modifications within these microbial consortia may further reinforce plant tolerance to subsequent herbivory through plant–soil feedback mechanisms (Gao et al., 2024). Thus, insect infestation represents a multifaceted stress that integrates plant metabolism, microbial interactions, and macromolecular defense processes (Friman et al., 2021). Despite growing evidence of these associations, the molecular basis linking plant metabolism and microbial dynamics during insect infestation remains poorly understood (Arkhipov et al., 2024; Li et al., 2024).

*Dendroctonus valens*, a major quarantine pest in China’s forestry sector, poses a severe threat to *Pinus tabuliformis*, resulting in the death of millions of pine trees across northern China (Sun et al., 2013; Yan et al., 2005). Native to North America, *D. valens* invades host phloem tissues, where aggregation of conspecifics facilitates colonization of the basal trunk and root regions, disrupting nutrient translocation and ultimately leading to tree mortality (Guérard et al., 2000; Raffa and Berryman, 1983; Sullivan, 2016; Wood, 1982). Increasing evidence indicates that the beetle’s success is closely linked to its microbial symbionts. *D. valens* forms a symbiotic invasive complex with *Leptographium procerum*, sustained through metabolic cooperation in which ammonia (NH₃) volatilized by bacterial partners accelerates fungal carbohydrate utilization and mitigates nutritional competition between the insect and its symbionts (Lu et al., 2010; Zhou et al., 2017). In addition, these associated microorganisms function as external biochemical degradation systems that detoxify host defensive compounds such as D-pinene, thereby enhancing beetle colonization (Liu et al., 2020). The gut microbiota further contributes to nutrient assimilation and metabolic regulation, maintaining the stability of this symbiotic relationship (Liu et al., 2024). Despite advances in understanding *D. valens*–microbe interactions, the molecular and macromolecular defense responses of *P. tabuliformis* during varying levels of infestation remain poorly characterized. In particular, little is known about how the phloem and rhizosphere microbiomes, together with their associated metabolites, coordinate the host’s resistance against *D. valens*.

In this study, we hypothesized that variations in microbial community composition and metabolite profiles correspond to differences in host resistance under distinct infestation intensities. To test this hypothesis, we integrated microbiome and metabolome analyses to characterize the phloem and rhizosphere responses of *P. tabuliformis*. Correlation analyses between key microbial taxa and defensive metabolites were conducted to elucidate potential functional interactions. These findings advance our understanding of the biochemical and macromolecular defense network of *P. tabuliformis*, providing a mechanistic foundation for developing microbe-assisted pest management and forest ecosystem restoration strategies.

## 2 Materials and methods

### 2.1 Insect collection and plant property

In August 2024, at the Longxing Forest Farm in Lvliang City, Shanxi Province (37.49808803°N, 111.60088638°E, with an average elevation of 1,400 m). According to the population density and phloem infested of *Pinus tabuliformis* by *Dendroctonus valens*, three individual were randomly selected for each of the following health categories: healthy (T0), mildly infested (T1), moderately infested (T2), and severely infested (T3) (Supplementary Table S1). For healthy *P. tabuliformis* trees, phloem tissues were collected at a height of 50 cm above the ground. For infested trees, phloem tissues were collected from the areas surrounding the galleries of *D. valens* (within 1 cm of the galleries). Simultaneously, root samples were collected at depths of 5 cm, 10 cm, and 15 cm from the base of each tree. Each set of root samples was placed into a 50 mL centrifuge tube. All samples were shipped to the laboratory via a dry shipper with dry ice.

Using sterile forceps, the root samples of *P. tabuliformis* were gently shaken in a laminar flow cabinet to remove loosely adherent soil particles. The remaining roots were then placed into a 50-mL centrifuge tube containing 20 mL of sterile PBS buffer. The tube was agitated to dislodge any additional loose soil. The suspension was subsequently centrifuged at 6,000 × g for 20 min at 4 °C to separate the rhizosphere soil particles and the supernatant. The supernatant was then filtered through a 0.22-μm sterile filter membrane to remove suspended particles and microbial cells, yielding a purified metabolite solution. Finally, all samples were stored at −80 °C for later microbiome and metabolomics analyses.

### 2.2 Microbiome sequencing and data assembly

DNA extraction from the phloem and rhizosphere soil of *P. tabuliformis* was carried out the E.Z.N.A.® soil DNA Kit (Omega Bio-tek, Norcross, GA, United States) according to the manufacturer’s instructions. For bacterial community, the bacterial 16S rRNA genes were amplified using the universal bacterial primers gene 338F (5’-ACTCCTACGGGAGGCAGCAG-3′) and 806R (5′- GGACTACHVGGGTWTCTAAT-3′) (Caporaso et al., 2011; Huse et al., 2008; Salas-González et al., 2021). For fungal community analysis, ITS sequences were amplified with primers ITS3F (5’-GCATCGATGAAGAACGCAGC-3′) and ITS4 (5’-TCCTCCGCTTATTGATATGC-3′) (White et al., 1990). The PCR mixture (20 μL) consisted of 10 ng DNA, 1 μL of 10 μM each primer, 2 μL of 2.5 mM dNTPs, 0.3 μL Fast Pfu polymerase, and 4 μL 5 × Fastpfu buffer. Thermal cycling comprised 3 min at 95 °C, then 35 cycles of 30 s at 95 °C, 30 s at 55 °C, and 45 s at 72 °C, ending with a 10 min extension at 72 °C. PCR was performed in triplicate with a 20-μL reaction mixture containing 10 μL of 2 × Phanta Max Master Mix, 0.8 μL of each primer (5 μmol/L), and 10 ng of template DNA. The resulting amplicons were extracted from 2% agarose gels, purified using the AxyPrep DNA Gel Extraction Kit (Axygen Biosciences, Union, CA, United States) per the manufacturer’s protocol, and quantified with Qubit 4.0 (Thermo Fisher Scientific, United States).

After demultiplexing, the resulting sequences were quality filtered with fastp (v0.19.6) (Magoč and Salzberg, 2011) and merged with FLASH (v1.2.7) (Chen et al., 2018). Then the high-quality sequences were de-noised using DADA2 (Edgar, 2013) plugin in the Qiime2 (Wang et al., 2007) (version 2020.2) pipeline with recommended parameters, which obtains single nucleotide resolution based on error profiles within samples. DADA2 denoised sequences are usually called amplicon sequence variants (ASVs) (Barberán et al., 2012). Sequences were rarefied to 6,000 per sample, achieving an average Good’s coverage of 97.90%. Taxonomic assignment was performed using the Naive Bayes classifier in Qiime2 with the SILVA 16S rRNA database (v138).

### 2.3 Microbiome analysis

To elucidate the microbial communities in the phloem and rhizosphere soil of *P. tabuliformis* under varying infestation levels, we analyzed bacterial and fungal communities in phloem (PT0, PT1, PT2, PT3) and rhizosphere soil (RT0, RT1, RT2, RT3). Bioinformatic analysis of the microbiota was carried out using the Majorbio Cloud platform (https://cloud.majorbio.com). Alpha diversity indices (observed ASV, Chao, ACE, Fisher, Shannon, Simpson) were used to assess genera richness and evenness. Chao and Shannon indices were selected for visualization. Alpha diversity differences were analyzed using the Kruskal-Wallis test, with *p* < 0.05 indicating significance. Beta diversity was assessed via principal co-ordinates analysis (PCoA), with euclidean distances representing dissimilarities. Relative abundance plots at the class and genus levels were used to compare microbial compositions. The relative abundances of fungi and bacteria in the phloem and rhizosphere soil of *P. tabuliformis* with varying degrees of infestation were compared using One-way ANOVA. Subsequently, post-hoc tests were conducted to identify differentially abundant microorganisms among the multiple groups.

To assess interactions between microbiota across varying infestation levels, multiple research approaches were used. These involved looking at correlations between infestation levels and microbial abundance, exploring relationships within the microbiome. Spearman correlation coefficients were applied to analyze pairwise co-occurrence of microbiota (bacteria and fungi) at class and genus levels.

### 2.4 Metabolome determination

To investigate metabolic changes in the phloem and rhizosphere soil of *P. tabuliformis* under different infestation levels, we conducted non-targeted metabolomics using liquid chromatography–mass spectrometry (LC–MS) on phloem (PT0, PT1, PT2, PT3) and rhizosphere soil (RT0, RT1, RT2, RT3) samples. After being frozen in liquid nitrogen for 15 min, metabolites were extracted from the samples. Analyses were done on a UHPLC-Q Exactive HF-X system (Thermo Fisher Scientific) in positive/negative ion modes. Quality control (QC) samples, made by mixing equal volumes of metabolites from all samples, assessed data quality and reproducibility. Experimental sample sequences were randomized to reduce instrument-related biases.

LC–MS raw data were preprocessed using Progenesis QI software (Waters Corporation) to generate a 3-dimensional data matrix in CSV format, including sample information, metabolite names, and spectral intensity. Metabolites were identified using HMDB, Metlin (https://metlin.scripps.edu/), and Majorbio databases. Features detected in ≥80% of samples were retained, with missing values imputed. Data were normalized by sum, and variables with a relative standard deviation >30% in QC samples were removed. Final data were log10-transformed for analysis.

### 2.5 Metabolome analysis

The similarity within groups and variability between groups for phloem (PT0, PT1, PT2, PT3) and rhizosphere soil (RT0, RT1, RT2, RT3) of *P. tabuliformis* were assessed via the Pearson correlation coefficient. Metabolite variation was measured via pairwise sample distances to form a distance matrix. A heatmap visualized these relations, verifying biological replicate consistency for differential metabolite analysis. Partial least squares discriminant analysis (PLS-DA) was employed to highlight differences in metabolite expression among samples.

Metabolites identified in positive and negative ion modes were compared against the Kyoto Encyclopedia of Genes and Genomes (KEGG). Compound database to classify them into hierarchical biological functions (superclass, class, subclass). Metabolite correlations were analyzed to identify metabolites with comparable expression trends, infer functional links, and suggest potential functions for unknown metabolites using Pearson’s r and Euclidean distance metrics. Differential metabolites between phloem and rhizosphere soil under different infestation levels were analyzed separately in positive, negative, and combined ion modes. Differential metabolites were selected based on VIP values ≥1 in the first two principal components of the orthogonal PLS-DA model, fold changes ≥1, and *p* < 0.05 using the Kruskal-Wallis test. Subsequent analysis utilized the merged ion mode. KEGG analysis was conducted on differential metabolites to clarify their metabolic pathways and biological impacts.

### 2.6 Association analysis of microbiome and metabolome

To determine the multiple correlations between the microbiome and metabolites in phloem and rhizosphere soil of *P. tabuliformis* and observe the changes in these metabolic functions when *D. valens* infested at different levels, we determined the multiple correlation relationships between the microbiome and metabolites. The relationships between bacteria and metabolites, and between fungi and metabolites, were analyzed. Initially, co-inertia analysis determined the global similarity between microbiome and metabolome samples, with correlations deemed significant at *p* < 0.05. Subsequently, to explore the distinct metabolic functions linked to differential microbes in phloem and rhizosphere soil of *P. tabuliformis* differential microbes and metabolites were identified through screening. Differential metabolites were identified using the same method as above, while differential bacteria were detected via group-wise relative abundance comparisons with the Kruskal-Wallis Test at a significance level of *p* < 0.05. Subsequently, the correlation between differential microbes and metabolites in the phloem and rhizosphere soil of *P. tabuliformis* at different infestation levels was calculated using Pearson correlation analysis.

## 3 Results

### 3.1 Microbial community shifts in response to different infestation levels in the phloem of *Pinus tabuliformis*

A subset of 12 samples from the phloem of *P. tabuliformis* was examined, and both bacterial and fungal sequencing were performed. Bacterial sequencing yielded 802,211 counts from 16S rRNA gene sequences, averaging 66,851 counts per sample, which translated into 2,445 ASVs across 29 phyla, 61 classes, 145 orders, 225 families, and 432 genera post-QC. Fungal sequencing, on the other hand, generated 746,313 counts from ITS gene sequences, averaging 62,193 counts per sample, leading to the identification of 622 ASVs belonging to 8 phyla, 28 classes, 58 orders, 127 families, and 180 genera after QC (Supplementary Table S2 and S3).

**Figure 1:**
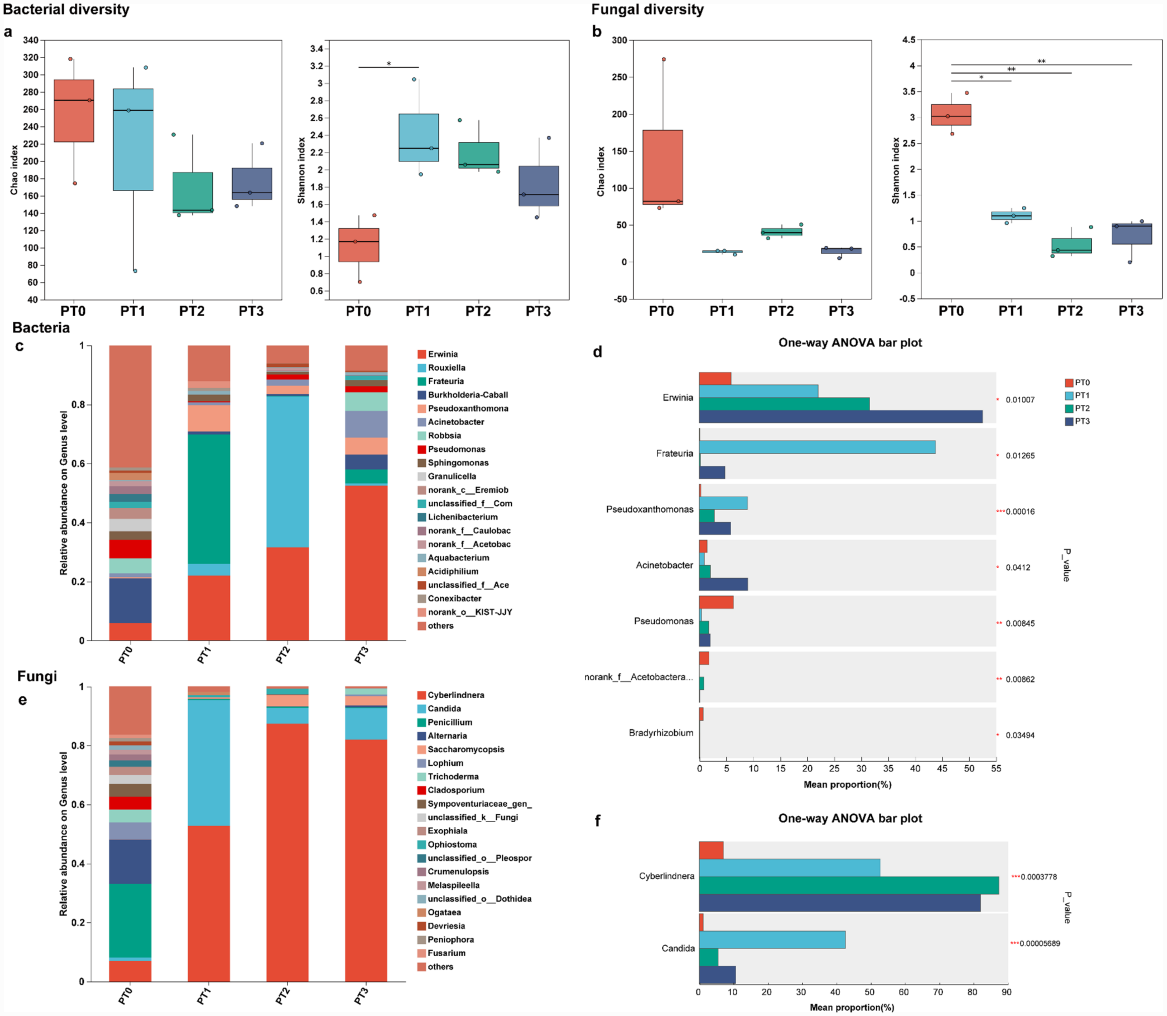
Change in microbiota composition and structure in the phloem of *Pinus tabuliformis* under different levels of infestation. Scatter plots show Chao and Shannon indices for bacterial **(a)** and fungal **(b)** diversity in phloem. Asterisks denote significant differences between groups (*n* = 3), *0.01 < *p* ≤ 0.05, **0.001 < *p* ≤ 0.01, ***p ≤ 0.001. Bar charts display bacterial **(c)** and fungal **(e)** community relative abundance at the genus level, highlighting the top 20 most abundant genera. Differences in abundance of dominant bacterial **(d)** and fungal **(f)** genera (One-way ANOVA, *p < 0.05, **p < 0.01, *** *p* < 0.001). PT0 denotes the phloem of healthy *Pinus tabuliformis*, PT1PT3 denotes the phloem of *Pinus tabuliformis* under different levels of infestation.

The diversity of bacteria and fungi was compared among samples from the phloem of *P. tabuliformis*. Analysis of alpha diversity metrics showed varying responses in bacterial and fungal diversity. In terms of bacterial diversity, PT0 displayed the highest richness (as indicated by the Chao index), followed by PT1, PT3, and PT2, while PT1 showed the highest evenness (measured by the Shannon index), followed by PT2, PT3, and PT0 (Figure 1a). For fungal diversity, PT0 also exhibited the highest richness, followed by PT2, PT3, and PT1, with PT0 also having the highest evenness, followed by PT1, PT3, and PT2 (Figure 1b). In short, after the healthy *P. tabuliformis* was infested, the diversity indices of both fungal and bacterial communities decreased, except for the Shannon index of bacteria (Figures 1a,b). In terms of beta diversity, PCoA revealed that among the four groups for both bacterial (Supplementary Figure S1a, Adonis, *R*^2^ = 0.09971, *p* = 0.005) and fungal samples (Supplementary Figure S1b, Adonis, *R*^2^ = 0.8169, *p* = 0.005), with PT2 showing partial overlap with PT3 in the bacterial community.

The microbial community structure of the phloem of *P. tabuliformis* was characterized at the class level. Across the four groups (PT0, PT1, PT2, and PT3), a diverse array of bacterial classes was observed, with Gammaproteobacteria being the dominant class in all groups (Supplementary Figure S1c). For fungi, Dothideomycetes was the most abundant class in PT0, while Saccharomycetes dominated PT1, PT2, and PT3 (Supplementary Figure S1d). At the genus level, the top five genera with an average abundance in PT0 were diverse, including *Burkholderia-Caballeronia-Paraburkholderia*, *Pseudomonas*, *Erwinia*, *Robbsia* and *Granulicella*. PT1 was dominated by *Frateuria* and *Erwinia*, with contributions from *Pseudoxanthomonas* and *Rouxiella*. PT2 was predominantly composed of *Rouxiella* and *Erwinia*, while PT3 had high abundances of *Erwinia*, *Acinetobacter*, *Robbsia*, *Pseudoxanthomonas* and *Burkholderia-Caballeronia-Paraburkholderia*. Notably, as the infestation level of *P. tabuliformis* increased, the abundance of *Erwinia* continuously rose, while the abundances of *Rouxiella* first increased and then decreased. The abundances of *Frateuria* and *Pseudoxanthomonas* significantly increased after healthy *P. tabuliformis* was infested (Figure 1c). The fungal community in PT0 was composed of several genera, with *Penicillium* and *Alternaria* being the most abundant, followed by *Cyberlindnera*, *Lophium* and *Cladosporium*. In contrast, PT1, PT2, and PT3 were dominated by *Cyberlindnera*, accompanied by varying levels of *Candida*. The abundance of *Cyberlindnera* initially increased and then decreased with the increase of infestation level in *P. tabuliformis*. After healthy *P. tabuliformis* was infested, the abundance of *Candida* significantly increased, while the abundances of *Penicillium* and *Alternaria* markedly decreased (Figure 1e).

In order to further understand whether the different levels of infestation by *Dendroctonus valens* have a significant impact on the composition of bacteria and fungi in the phloem of *P. tabuliformis*, One-way ANOVA analysis was carried out in all collected samples: at the genus level, the composition of bacteria (Supplementary Table S4) and fungi (Supplementary Table S5) in phloem showed significant differences according to the different levels of infestation (*p* < 0.05). In the bacterial community, *Erwinia*, *Frateuria*, *Pseudoxanthomonas*, *Acinetobacter*, *Pseudomonas*, *Norrank _ f _ Acetobacteraceae* and *Bradyrhizobium* showed significant difference abundance (*p* < 0.05, Figure 1d). In the fungal community, both *Cyberlindnera* and *Candida* showed significant difference in abundance (*p* < 0.05, Figure 1f).

### 3.2 Microbial community shifts in response to varying infestation levels in the rhizosphere soil

The microbiome sequencing of rhizosphere soil samples collected from *P. tabuliformis* was analyzed similarly. Bacterial sequencing revealed 700,112 counts from 16S rRNA gene sequences of 12 samples, averaging 58,343 counts per sample, with 19,981 ASVs spanning 41 phyla, 111 classes, 267 orders, 438 families, and 864 genera post-QC. Fungal sequencing produced 869,185 counts from ITS gene sequences, averaging 72,432 counts per sample, resulting in 4,848 ASVs distributed across 16 phyla, 53 classes, 125 orders, 283 families, and 505 genera post-QC (Supplementary Tables S6, S7).

**Figure 2:**
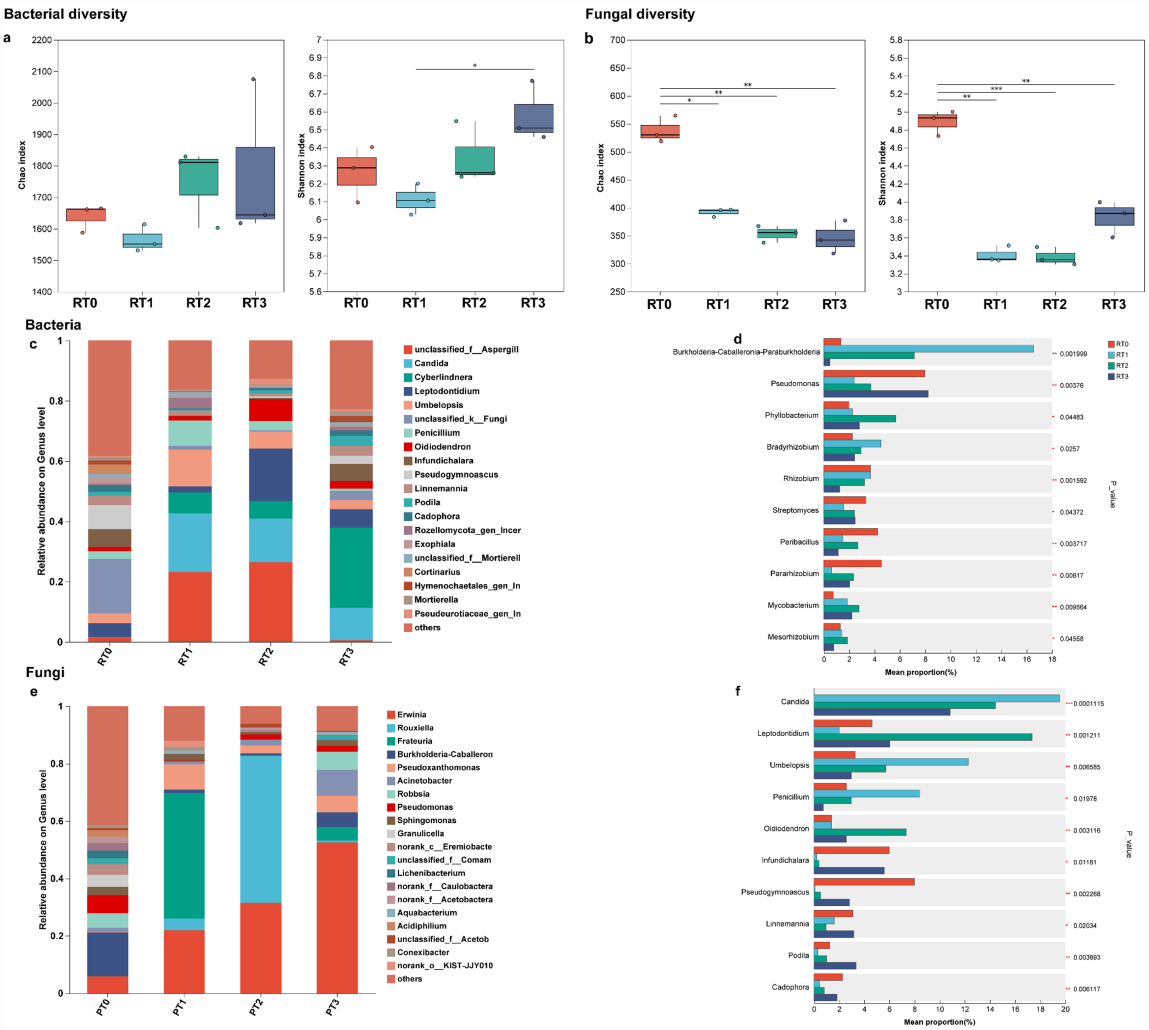
Change in microbiota composition and structure in the rhizosphere soil of *Pinus tabuliformis* under different levels of infestation. Scatter plots show Chao and Shannon indices for bacterial **(a)** and fungal **(b)** diversity in rhizosphere soil. Asterisks denote significant differences between groups (*n* = 3), *0.01 < *p* ≤ 0.05, **0.001 < *p* ≤ 0.01, ***p ≤ 0.001. Bar charts display bacterial **(c)** and fungal **(e)** community relative abundance at the genus level, highlighting the top 20 most abundant genera. Differences in abundance of dominant bacterial **(d)** and fungal **(f)** genera (one-way ANOVA, *p < 0.05, **p < 0.01, *** *p* < 0.001). RT0 denotes rhizosphere soil of healthy *Pinus tabuliformis*, RT1RT3 denotes rhizosphere soil of *Pinus tabuliformis* under different levels of infestation.

The diversity of bacteria and fungi was compared among samples from the rhizosphere soil of *P. tabulaeformi*. Analysis of alpha diversity indices indicated varied responses in both bacterial and fungal diversity under different levels of infestation. For bacterial diversity, the numerical values of richness (as indicated by the Chao index) were similar in RT0, RT2 and RT3, with RT1 being the lowest, and the numerical values of evenness showed similar in RT0 and RT2, with RT1 being the lowest, RT3 being the highest (Figure 2a). For fungal diversity, RT0 exhibited the highest richness and evenness (Figure 2b). In terms of beta diversity, PCoA demonstrated significant separation among the four groups for both bacterial (Supplementary Figure S2a, Adonis, *R*^2^ = 0.8858, *p* = 0.0002) and fungal (Supplementary Figure S2b, Adonis, *R*^2^ = 1, *p* = 0.0002) samples, with significant aggregation within each group’s samples.

The microbial community structure of the rhizosphere soil of *P. tabuliformis* was also characterized at the class level. Across the four soil samples (RT0, RT1, RT2, and RT3), a diverse spectrum of bacterial classes was identified. In RT0, RT1, RT2 and RT3, Alphaproteobacteria, Gammaproteobacteria, Bacilli, Actinobacteria and Vicinamibacteria, and RT2 had a high abundance of Acidobacteriae (Supplementary Figure S2c). For fungi, Leotiomycetes, unclassified_k__Fungi, Agaricomycetes, Dothideomycetes and Eurotiomycetes were the dominant classes in RT0. RT1 and RT2 exhibited a high abundance of Eurotiomycetes, Saccharomycetes, along with Umbelopsidomycetes and Leotiomycetes. Saccharomycetes, Leotiomycetes and Mortierellomycetes being the most abundant in RT3 (Supplementary Figure S2d). At the level of identified genera, the microbial community composition of the rhizosphere soil of *P. tabuliformis* also varied significantly among the samples. Within the bacterial community, RT0 was characterized by a diverse array of genera, including *Pseudomonas*, *Pararhizobium*, *Peribacillus*, *Rhizobium*, *Streptomyces* and *Bradyrhizobium*. RT1 showed a high abundance of *Burkholderia-Caballeronia-Paraburkholderia*, *Bradyrhizobium*, *Rhizobium*, *Pseudomonas* and *Phyllobacterium*, RT2 was predominantly populated by members of the *Burkholderia-Caballeronia-Paraburkholderia*, *Phyllobacterium*, *Pseudomonas*, *Rhizobium* and *Bradyrhizobium*, RT3 had a high abundance of *Pseudomonas*, *Phyllobacterium*, *Streptomyces*, *Bradyrhizobium*, *Mycobacterium* and *Pararhizobium*. With the increase of infested level in *P. tabuliformis*, the abundance of *Bradyrhizobium* first increased and then decreased, while that of *Pseudomonas* first decreased and then increased (Figure 2c). Within the fungal community, RT0 was characterized by a diverse assemblage, with *unclassified_k__Fungi* being the most abundant genus, followed by *Pseudogymnoascus*, *Infundichalara*, *Leptodontidium*, *Umbelopsis*, *Cortinariu* and *Linnemannia*. RT1 exhibited a more even distribution of genera, with high abundances of *unclassified_f__Aspergillaceae*, *Candida*, *Umbelopsis*, *Penicillium*, *Cyberlindnera* and *Leptodontidium*. RT2 was dominated by *unclassified_f__Aspergillaceae*, *Leptodontidium* and *Candida*, with contributions from *Oidiodendron*, *Cyberlindnera* and *Umbelopsis*. RT3 showed a similar pattern, with *Cyberlindnera* and *Candida* being the most abundant genera, followed by *Leptodontidium*, *Infundichalara* (5.64%), *Podila*, *Linnemannia* and *Umbelopsis*. With the increase of infested level in *P. tabuliformis*, the abundance of *Penicillium* and *Umbelopsis* first increased and then decreased, while that of *Infundichalara*, *Pseudogymnoascus and Linnemannia* first decreased and then increased. It was worth noting that *Candida* and *Penicillium* were found in the rhizosphere soil of healthy *P. tabuliformis* (Figure 2d).

**Figure 3:**
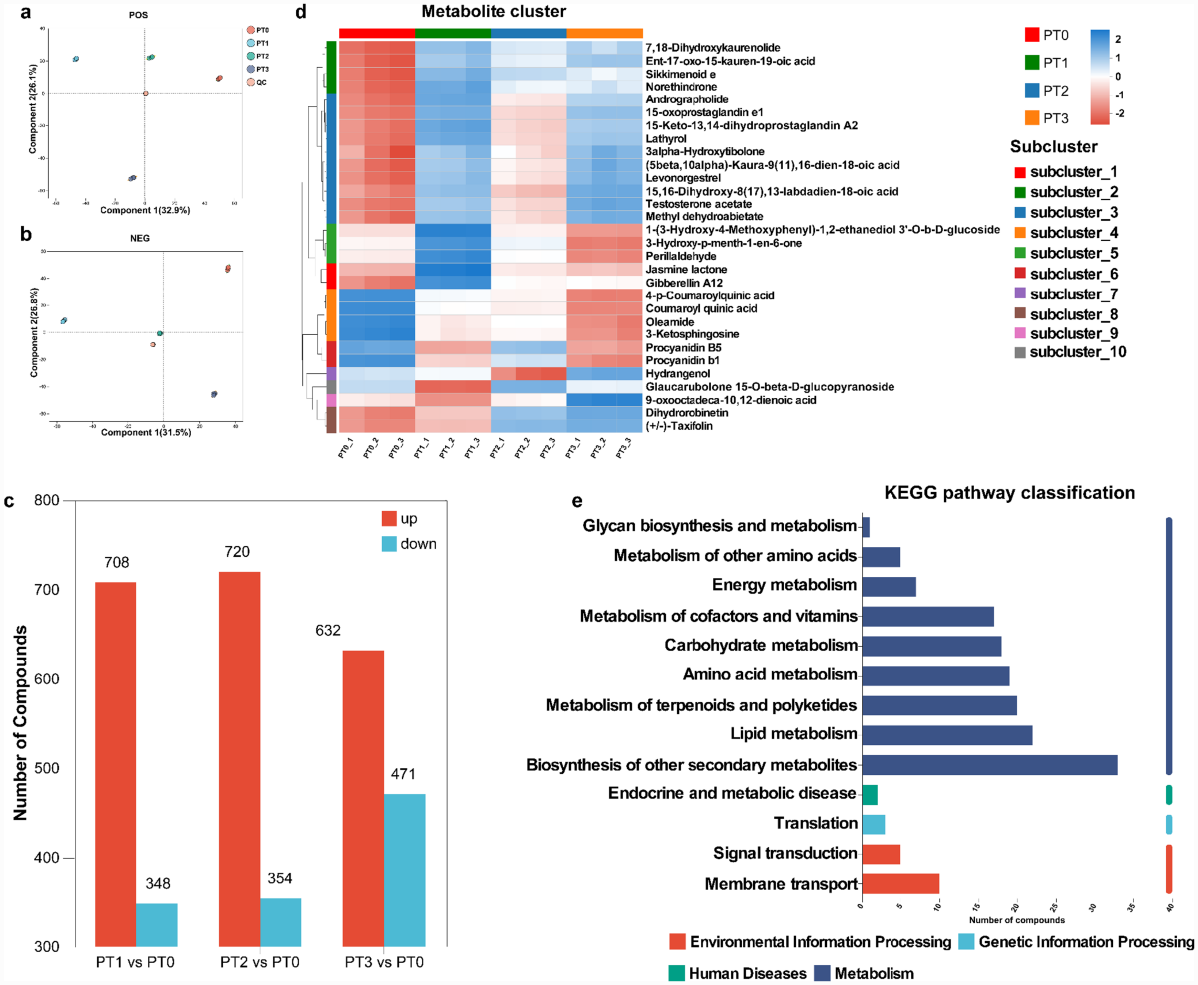
Overview of metabolites identified by untargeted metabolomics profiling and differentially accumulated metabolites in the phloem of *Pinus tabuliformis* under different levels of infestation. Partial least squares discriminant analysis showed the clustering of differential metabolites in positive ion mode **(a)** and negative ion mode **(b)**, with the coordinate axis indicating each principal component’s contribution to total variance. **(c)** Differential metabolite count. A heatmap showed differential metabolite clustering **(d)** in phloem of *Pinus tabuliformis*. Kyoto Encyclopedia of Genes and Genomes (KEGG) pathway classification **(e)** covered differential metabolites in phloem of *Pinus tabuliformis*. POS denotes positive ion mode, NEG denotes negative ion mode. PT0 denotes phloem of healthy *Pinus tabuliformis*, PT1–PT3 denotes phloem of *Pinus tabuliformis* at different levels of infestation.

In order to further understand whether the different levels of infestation by *D. valens* have a significant impact on the composition of bacteria and fungi in the rhizosphere soil of *P. tabuliformis*, One-way ANOVA analysis was carried out in all collected samples: at the genus level, the composition of bacteria (Supplementary Tables S8) and fungi (Supplementary Tables S9) in rhizosphere soil showed significant differences according to the different levels of infestation (*p* < 0.05). In the bacterial community, *Burkholderia-Caballeronia-Paraburkholderia*, *Pseudomonas*, *Phyllobacterium*, *Bradyrhizobium*, *Rhizobium*, *Streptomyces*, *Peribacillus*, *Pararhizobium*, *Mycobacterium* and *Mesorhizobium* showed significant difference abundance (*p* < 0.05 Figure 3d). In the fungal community, *Candida*, *Leptodontidium*, *Umbelopsis*, *Penicillium*, *Oidiodendron*, *Infundichalara*, *Pseudogymnoascus*, *Linnemannia*, *Podila* and *Cadophora* showed significant difference in abundance (*p* < 0.05, Figure 2f).

### 3.3 Dynamic metabolites in phloem of *Pinus tabuliformis* in response to infestion levels of *Pinus tabuliformis*

The metabolites in the phloem of *P. tabuliformis* were detected by LC–MS under different levels of infestation of *D. valens*. Conduct a series of preprocessing steps on the raw data, which primarily include filtering out missing values, imputing missing values, normalizing the data, QC verification, and data transformation.

The metabolite output in phloem of *P. tabuliformis* under different levels of infestation was categorized into two groups: positive and negative ion modes (Supplementary Figures S5, S6). In positive ion mode, 7,839 compounds were detected, with 1,397 identified post - QC. In negative ion mode, 8,131 compounds were detected, with 1,458 identified post - QC. Overall, 2,855 metabolites were identified for further analysis in the combined positive and negative ion mode (Table 1).

**Table 1:** Statistics for total and identified ion number in phloem of *Pinus tabuliformis*.

Ion mode	All peaks	Identified metabolites	Metabolites in library	Metabolites in KEGG
Positive	7,839	1,397	894	449
Negative	8,131	1,458	1,141	512
Positive and negative	15,970	2,855	2035	961

The metabolites in the phloem of *P. tabuliformis* under different infestation levels have been studied. Results indicated significant clustering of QC samples in PLS-DA plots for both ion modes, confirming the reliability of metabolic data (Figures 3a,b). Samples from the phloem of *P. tabuliformis* under different infestation levels clustered within their respective groups and showed marked separation among the four groups in the PLS-DA plot, reflecting significant metabolic variations. After QC, PT1, PT2, and PT3 exhibited varying numbers of up-regulated and down-regulated metabolites compared to PT0 (Figure 3c).

The metabolite changed in the phloem of *P. tabuliformis* under different infestation levels, a combination of positive and negative ion modes was used for subsequent analysis. In positive and negative ion mode, 15,970 compounds were detected, with 2,855 identified. Among these, 1,150 metabolites were found to have significant differences, with ion concentrations significantly varying among the four groups (Figure 3d). Significant positive and negative correlation relationships were observed among the different metabolites (Supplementary Figure S5). KEGG pathway classification revealed that the significantly different metabolites were primarily categorized into glycan biosynthesis and metabolism, metabolism of cofactors and vitamins, amino acid metabolism, metabolism of terpenoids and polyketides, lipid metabolism, metabolism of secondary metabolites, and signaling transduction (Figure 3e).

The differential metabolites identified by comparing PT1, PT2, and PT3 with PT0, respectively, were subjected to KEGG enrichment pathway analysis, revealing enrichment in the following six pathways: plant hormone signal transduction, galactose metabolism, phenylpropanoid biosynthesis, stilbenoid, diarylheptanoid, and gingerol biosynthesis, isoleucine biosynthesis, tyrosine metabolism, alpha-linolenic acid metabolism, and flavonoid biosynthesis. Specifically, the enrichment results for PT1 vs. PT0 and PT3 vs. PT0 were predominantly in both phenylpropanoid biosynthesis and flavonoid biosynthesis, while those for PT2 vs. PT0 were mainly in flavonoid biosynthesis (Supplementary Figure S6). These findings demonstrate that there are significant differences in the enrichment of metabolites in the phloem of *P. tabuliformis* under different levels of infestation caused by *D. valens*. Moreover, the phloem of *P. tabuliformis* plays a more important role in phenylpropanoid biosynthesis and flavonoid biosynthesis pathways during the invasion of *D. valens*.

**Figure 4:**
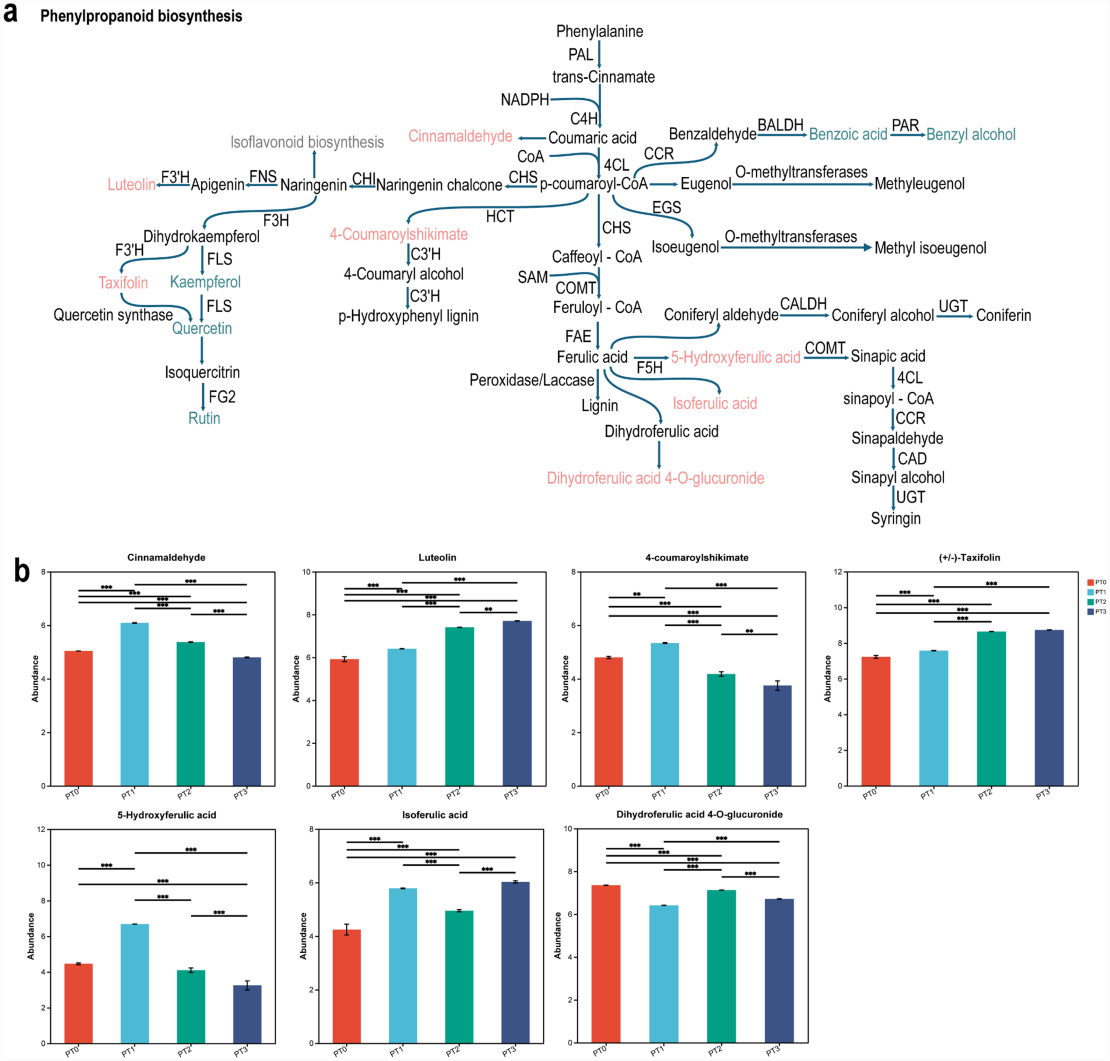
Differential response of phenylpropanoid biosynthesis and branching pathways in the phloem of *Pinus tabuliformis* under different levels of infestation. **(a)** The putative phenylpropanoid biosynthesis pathway of *Pinus tabuliformis* reconstructed from the reference Kyoto Encyclopedia of Genes and Genomes (KEGG) pathway. **(b)** Box plots show the relative content of metabolites from the phenylpropanoid pathway indifferent sample types *Pinus tabuliformis*. One-way ANOVA was used for statistical tests between comparison groups. *p < 0.05, ** *p* < 0.01, *** *p* < 0.001. PT0 denotes phloem of healthy *Pinus tabuliformis*, PT1PT3 denotes phloem of *Pinus tabuliformis* at different levels of infestation.

A comparative metabolome analysis indicated that *D. valens* infestation affected the biosynthesis of phenylpropanoids and compounds deriving from this pathway (Figure 4). The relative contents of some metabolites in phenylpropionic acid biosynthesis pathway in samples showed significant differences under different infestation levels, such as cinnamaldehyde, luteolin and 4-coumaroylshikimate, and derivatives of benzoic acid, benzyl alcohol and kaempferol (Figure 4a). Luteolin and taxifolin increased significantly with the increase of infestation degree of *P. tabuliformis*. Additionally, after *P. tabuliformis* was infested, cinnamaldehyde, 4-coumaroylshikimate and 5-hydroxyferulic acid decreased significantly with the increase of infestation level (Figure 4b). In addition, the derivatives of metabolites in phenylpropanoid biosynthesis pathway changed significantly after *P. tabuliformis* was infested by *D. valens* (Figure 4b). However, these substances are involved in plants, such as 2,5-dihydroxybenzaldehyde, Kaempferol 3-O-arabinoside, Kaempferol 3-O-rhamnoside-7-O-rhamnoside and, Kaempferol 3-O-rhamnoside-7-O-rhamnoside and so on.

### 3.4 Dynamic metabolites in rhizosphere soil of *Pinus tabuliformis* in response to infestion levels

The metabolites in the rhizosphere soil of *P. tabuliformis* were detected by LC–MS under different levels of infestation of *D. valens*. Conduct a series of preprocessing steps on the raw data, which primarily include filtering out missing values, imputing missing values, normalizing the data, QC verification, and data transformation.

The metabolite output in the rhizosphere soil of *P. tabuliformis* at different health level of *D. valens* was categorized into two groups: positive and negative ion modes (Supplementary Figures S7, S8). In positive ion mode, 4,357 compounds were detected, with 925 identified post - QC. In negative ion mode, 2,677 compounds were detected, with 729 identified post - QC. Overall, 1,171 metabolites were identified for further analysis in the combined positive and negative ion mode (Table 2).

**Table 2:** Statistics for total and identified ion number in rhizosphere soil of *Pinus tabuliformis*.

Ion mode	All peaks	Identified metabolites	Metabolites in library	Metabolites in KEGG
Positive	4,357	925	624	328
Negative	2,677	729	547	281
Positive and negative	7,034	1,654	1,171	609

**Figure 5:**
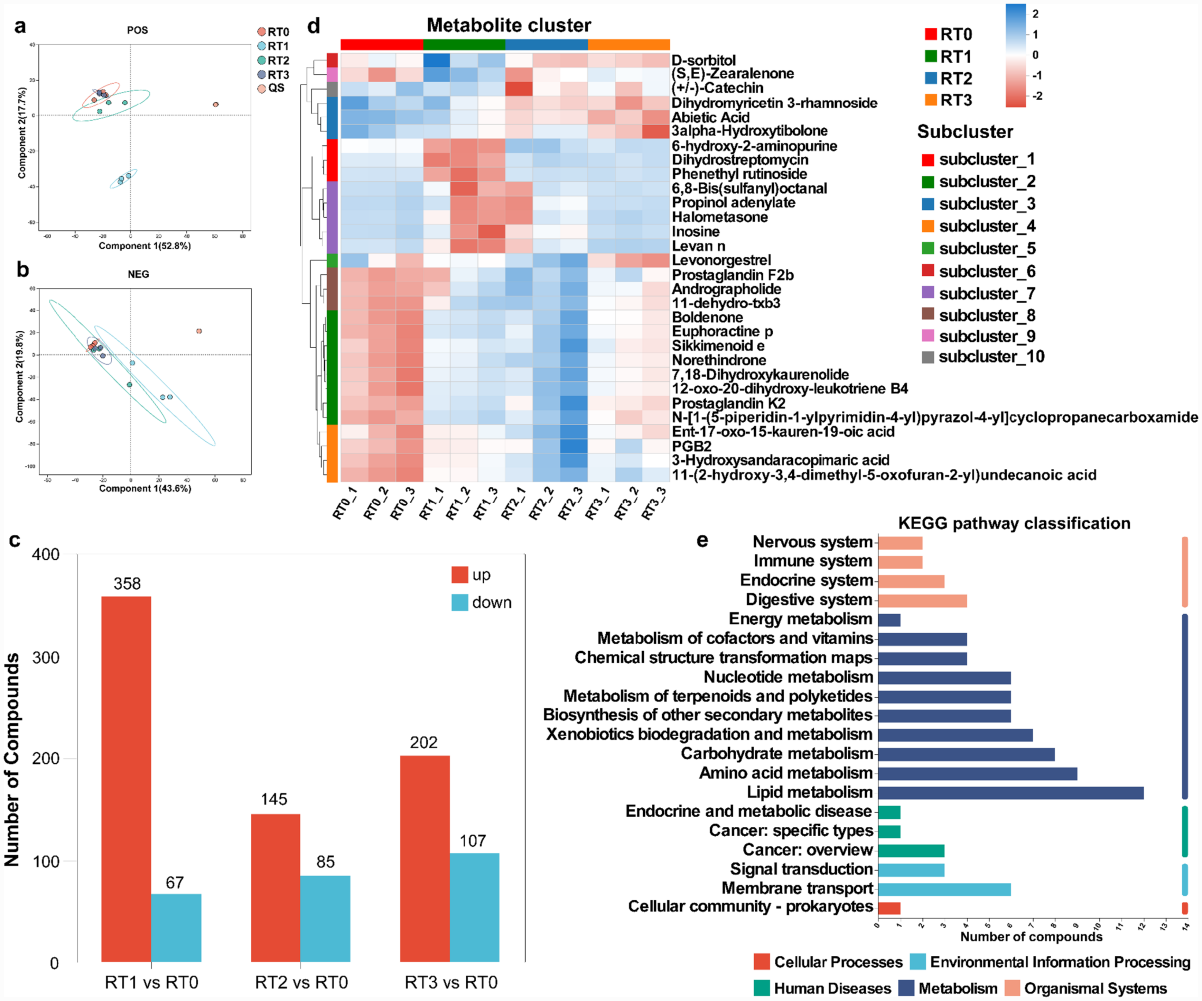
Overview of metabolites identified by untargeted metabolomics profiling and differentially accumulated metabolites in the rhizosphere soil of *Pinus tabuliformis* under different levels of infestation. Partial least squares discriminant analysis showed the clustering of differential metabolites in positive ion mode **(a)** and negative ion mode **(b)**, with the coordinate axis indicating each principal component’s contribution to total variance. **(c)** Differential metabolite count. A heatmap showed differential metabolite clustering **(d)** in rhizosphere soil of *Pinus tabuliformis*. Kyoto Encyclopedia of Genes and Genomes (KEGG) pathway classification **(e)** covered differential metabolites in rhizosphere soil of *Pinus tabuliformis*. POS denotes positive ion mode, NEG denotes negative ion mode. RT0 denotes rhizosphere soil of healthy *Pinus tabuliformis*, RT1–RT3 denotes rhizosphere soil of *Pinus tabuliformis* at different levels of infestation.

The metabolites in the rhizosphere soil of *P. tabuliformis* under different infestation levels have been studied. Results indicated significant clustering of QC samples in PLS-DA plots for both ion modes, confirming the reliability of metabolic data (Figures 5a,b). Samples from the rhizosphere soil of *P. tabuliformis* at different levels of infestation groups were significantly clustered in RT1 and RT2 groups, respectively, indicating that there were significant differences in metabolite profiles between them, while RT0 and RT3 overlapped, indicating that the metabolic reactions between them were similar in positive ion modes, and samples RT0, RT2 and RT3 overlapped, indicating similar metabolite profiles between them in negative ion modes. After QC, compared with RT0, there are different amounts of up-regulated and down-regulated metabolites in RT1, RT2 and RT3, respectively, (Figure 5c).

The metabolite changed in the rhizosphere soil of *P. tabuliformis* under different infestation levels, a combination of positive and negative ion modes was utilized for subsequent analysis. In positive and negative ion mode, 7,034 compounds were detected, with 2,855 identified. Among these, 312 metabolites were found to have significant differences, with ion concentrations significantly varying among the four groups (Figure 5d). Significant positive and negative correlation relationships were observed among the different metabolites (Supplementary Figure S9). KEGG pathway classification revealed that the significantly different metabolites were primarily categorized into immune system, metabolism of cofactors and vitamins, metabolism of terpenoids and polyketides, metabolism of secondary metabolites, amino acid metabolism, lipid metabolism, and signaling transduction (Figure 5e).

The differential metabolites identified by comparing RT1, RT2, and RT3 with RT0, respectively, were subjected to KEGG enrichment pathway analysis, revealing enrichment in the following six pathways, including steroid hormone biosynthesis, steroid degradation, intestinal immune network for lgA production, small cell lung cancer, th17 cell differentiation, and gastric cancer. Compared with RT0, they are mainly enriched in steroid hormone biosynthesis pathway (Supplementary Figure S10). These results showed that the enrichment results of metabolites in rhizosphere soil of *P. tabuliformis* were similar under different levels of infestation caused by *D. valens*. Moreover, the rhizosphere soil of *P. tabuliformis* plays a more important role in steroid hormone biosynthesis pathway during the invasion of *D. valens*.

**Figure 6:**
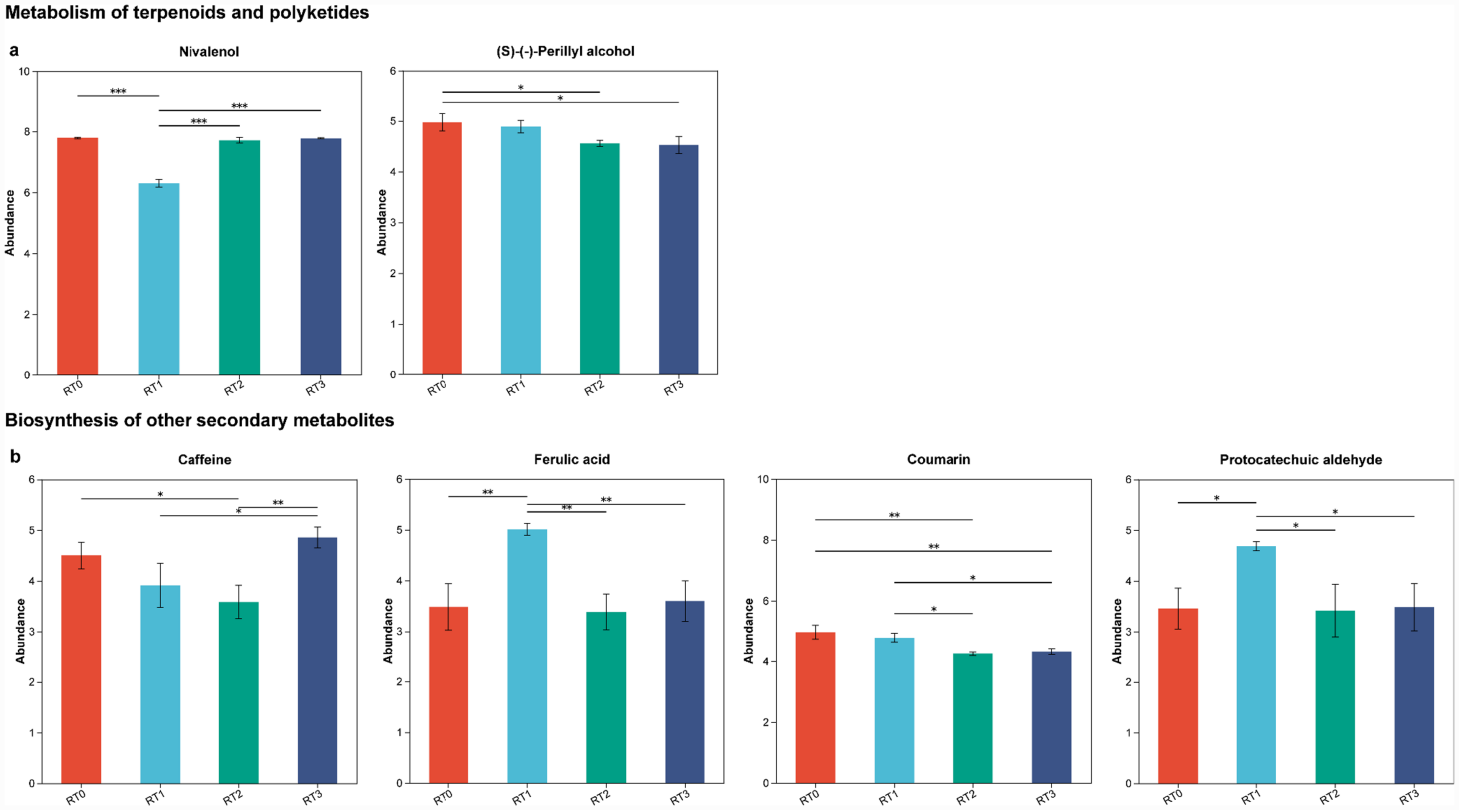
Differential response of metabolism of terpenoids and polyketides and the biosynthesis of other secondary metabolites in the rhizosphere soil of *Pinus tabuliformis* under different levels of infestation. Box plots show the relative content of metabolites from the metabolism of terpenoids **(a)** and polyketides and the biosynthesis of other secondary metabolites **(b)** indifferent sample types *Pinus tabuliformis*. One-way ANOVA was used for statistical tests between comparison groups. *p < 0.05, ** *p* < 0.01, *** *p* < 0.001. RT0 denotes rhizosphere soil of healthy *Pinus tabuliformis*, RT1–RT3 denotes rhizosphere soil of *Pinus tabuliformis* at different levels of infestation.

A comparative metabolome analysis indicated that *D. valens* infestation affected the metabolism of terpenoids and polyketides, as well as the biosynthesis of other secondary metabolites in the rhizosphere soil. Significant differences were observed in the levels of specific metabolites under varying levels of infestation by *D. valens*. Within the category of metabolism of terpenoids and polyketides, compounds such as nivalenol and (S)-(−)-perillyl alcohol exhibited notable variations (Figure 6a). Similarly, within the biosynthesis of other secondary metabolites, metabolites including caffeine, ferulic acid, coumarin and protocatechuic aldehyde also showed significant differences (Figure 6b).

### 3.5 Association analysis revealed the microbial function in phloem of *Pinus tabuliformis* under different levels of infestation

**Figure 7:**
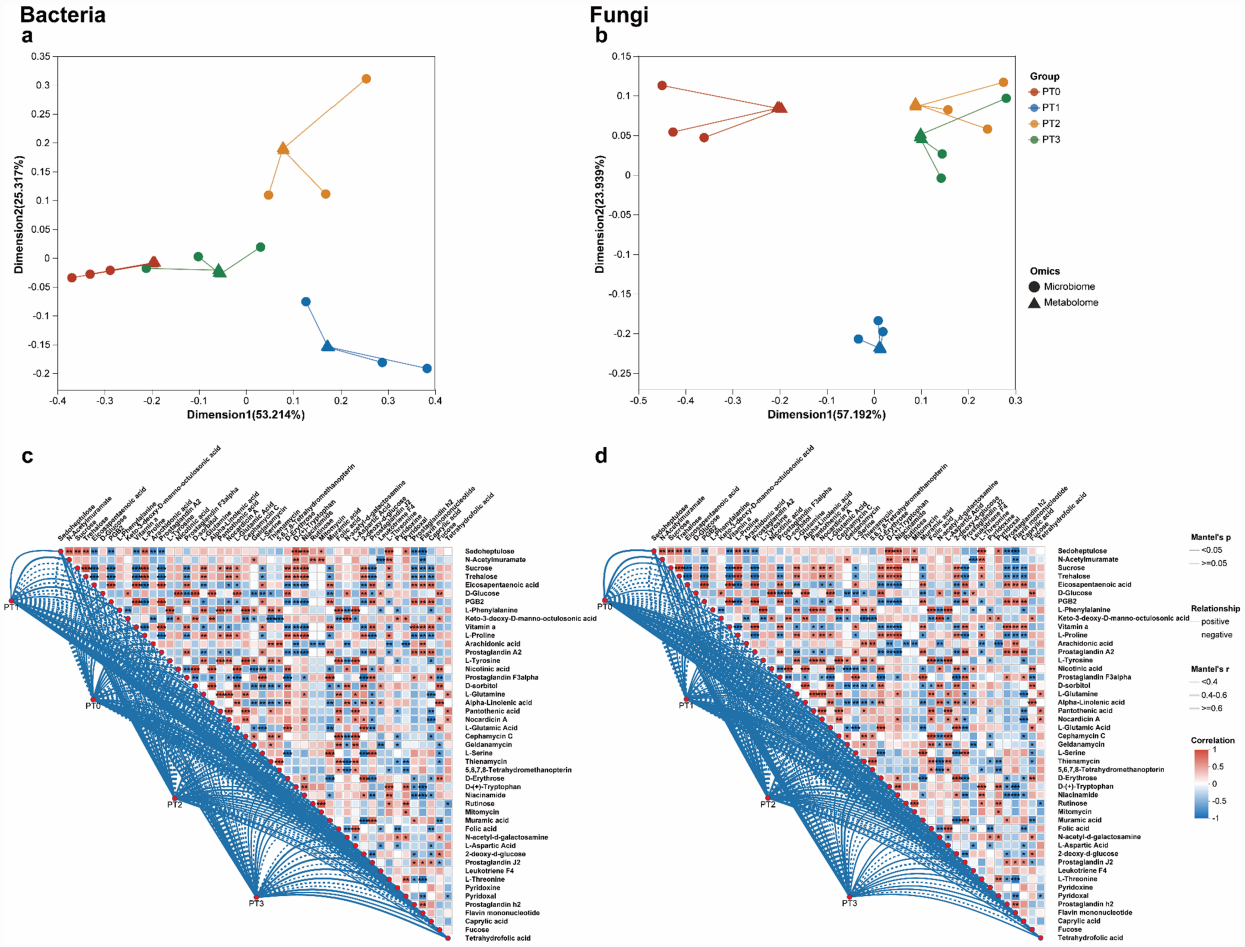
Correlation between microbes and metabolites in phloem of *Pinus tabuliformis* under different levels of infestation. Global similarity between microbiome and metabolome samples of phloem [bacteria **(a)** or fungi **(b)**], respectively. Each data point denotes an individual sample with distinct shapes. Lines signify the association between microbiome and metabolome. Mantel test evaluating the correlations between differential microorganisms and metabolites in phloem for bacterial **(c)** and fungal **(d)** communities. PT0 denotes phloem of healthy *Pinus tabuliformis*, PT1–PT3 denotes phloem of *Pinus tabuliformis* at different levels of infestation.

To explore the relationship between microbial physiological functions and biological phenotypes in the phloem of *P*. *tabulaeformis* under different infestation levels, an integrative analysis of microbes and metabolites was conducted. Co-inertia analysis was utilized to demonstrate a substantial and significant correlation between bacterial and fungal communities, on one hand, and the metabolite profiles derived from both the phloem (Figures 7a,b) of *P*. *tabulaeformis*.

Mantel correlation analyses were conducted to explore how shifts in microbial communities relate to changes in key differential metabolites in the phloem of *Pinus tabuliformis* under different infestation levels. In the bacterial network, only a few weak correlations were detected at PT0, primarily associated with basic carbohydrates such as sedoheptulose, sucrose, trehalose, and D-glucose. At PT1 and PT2, correlation density increased markedly, with enriched associations involving amino acids (e.g., L-phenylalanine, L-proline, L-tyrosine, L-glutamine) and lipid-derived metabolites including arachidonic acid and eicosapentaenoic acid. At PT3, the bacterial network reached its highest complexity, with numerous strong correlations (Mantel’s r ≥ 0.6) involving prostaglandins (e.g., prostaglandin A2, F3alpha, J2), antimicrobial-related metabolites (e.g., nocardicin A, Geldanamycin, mitomycin), and multiple vitamins or cofactors (e.g., pantothenic acid, pyridoxine, tetrahydrofolic acid) (Figure 7c). The fungal network exhibited a similar but more pronounced trend. At PT0, only weak correlations were observed, concentrated in primary carbohydrate metabolism. Compared with PT1, both the number and strength of correlations increased substantially at PT2, with enriched associations involving amino acids and fatty acid–derived metabolites such as arachidonic acid and eicosapentaenoic acid. At PT3, the fungal network displayed the greatest complexity, characterized by numerous strong correlations (Mantel’s r ≥ 0.6) with prostaglandins, antimicrobial compounds (e.g., nocardicin A, Geldanamycin), and vitamin-related metabolites (Figure 7d).

**Figure 8:**
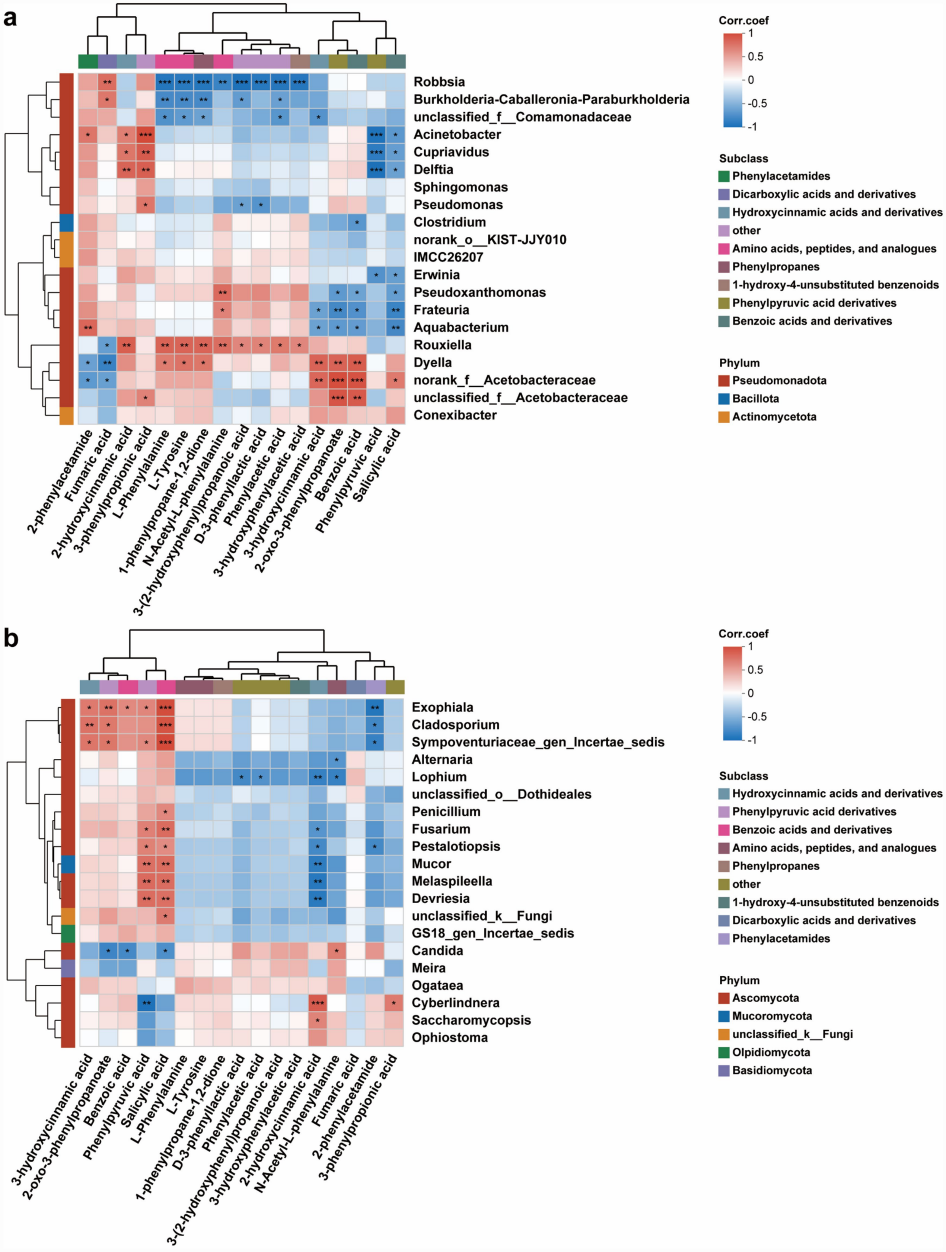
Correlation analysis between differential microorganisms and key metabolites associated with major metabolic pathways in the phloem of *Pinus tabuliformis* under different infestation levels. Spearman correlation heatmaps depicting the associations between differential bacterial **(a)** and fungal **(b)** taxa and key metabolites enriched in major metabolic pathways. Red and blue colors denote positive and negative correlations, respectively, with asterisks indicating levels of statistical significance (*p* < 0.05, *p* < 0.01, *p* < 0.001). Metabolites are grouped by subclass, and bacterial taxa are annotated by phylum. PT0 denotes phloem of healthy *Pinus tabuliformis*, PT1-PT3 denotes phloem of *Pinus tabuliformis* at different levels of infestation.

Correlation analysis revealed distinct interaction patterns between differential bacterial taxa and phenylpropanoid pathway metabolites. Several Pseudomonadota genera, including *Robbsia*, *Burkholderia-Caballeronia-Paraburkholderia*, *Acinetobacter*, and *Cupriavidus*, exhibited significant positive correlations with upstream phenylpropanoid intermediates such as 2-phenylacetamide, fumaric acid, and 1-phenylpropane-1,2-diol. In contrast, taxa such as *Dyella*, *Aquabacterium*, and members of Acetobacteraceae showed strong negative correlations with compounds including benzoic acid, salicylic acid, and phenylpyruvic acid (Figure 8a). The fungal network displayed a complementary but more pronounced correlation structure. Yeast genera such as *Candida*, *Meira*, *Ogataea*, *Cyberlindnera*, and *Saccharomycopsis* were positively correlated with upstream phenylpropanoid intermediates (e.g., 3-hydroxycinnamic acid, phenylpyruvic acid, L-phenylalanine). Conversely, filamentous fungi including *Cladosporium*, *Exophiala*, *Penicillium*, and taxa within Dothideales and Pleosporales showed consistent negative correlations with downstream metabolites such as 3-phenylpropionic acid and 2-phenylacetamide (Figure 8b).

### 3.6 Association analysis revealed the microbial function in rhizosphere soil of *Pinus tabuliformis* under different levels of infestation

**Figure 9:**
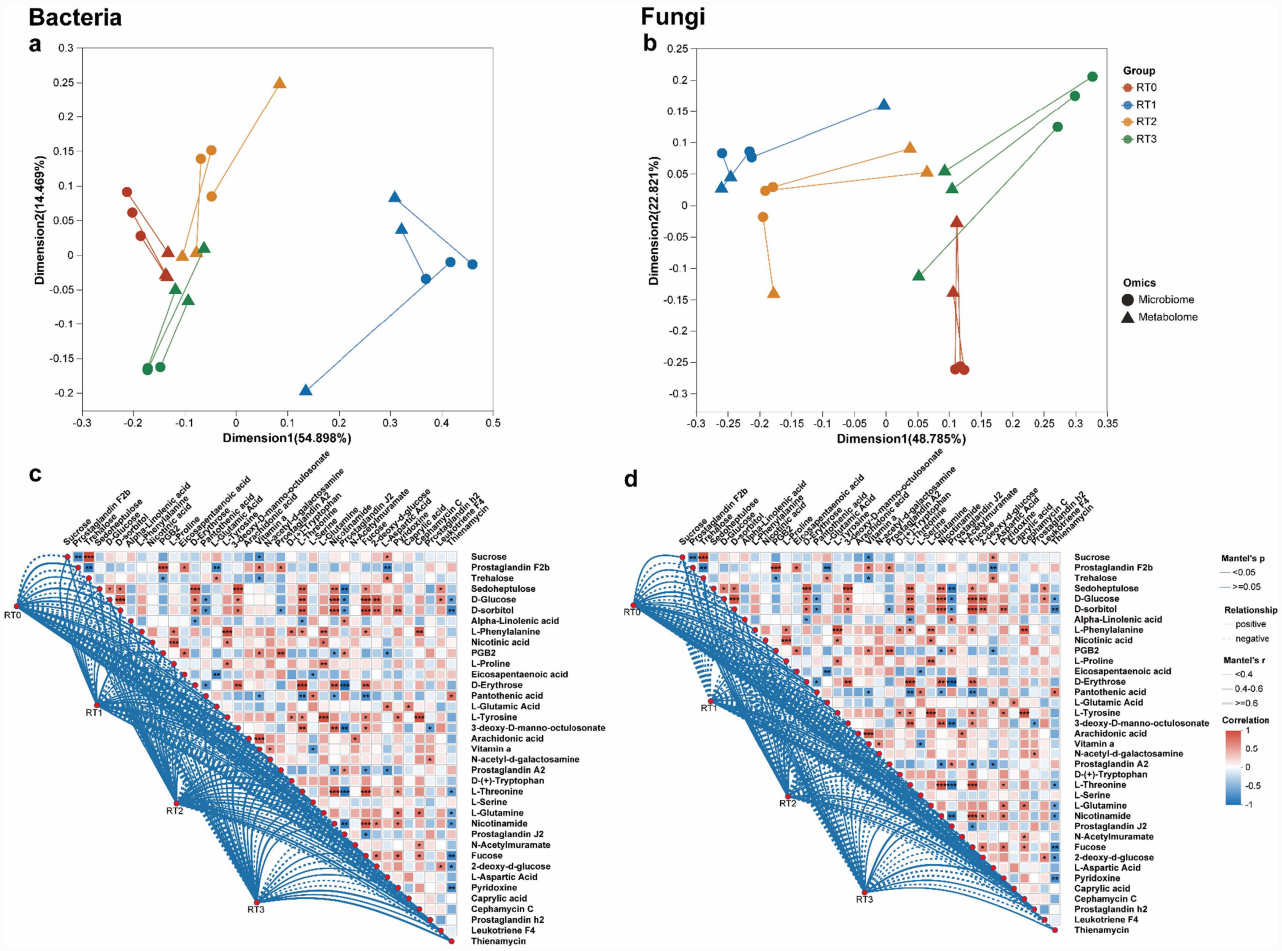
Correlation between microorganisms and metabolites in rhizosphere soil of *Pinus tabuliformis* under different levels of infestation. Global similarity between microbiome and metabolome samples of rhizosphere soil [bacteria **(a)** or fungi **(b)**], respectively. Each data point denotes an individual sample with distinct shapes. Lines signify the association between microbiome and metabolome. Mantel test evaluating the correlations between differential microorganisms and metabolites in hizosphere soil for bacterial **(c)** and fungal **(d)** communities. RT0 denotes rhizosphere soil of healthy *Pinus tabuliformis*, RT1–RT3 denotes rhizosphere soil of *Pinus tabuliformis* at different levels of infestation.

To explore the relationship between microbial physiological functions and biological phenotypes in the rhizosphere soil of *P. tabuliformis* under different infestation levels, an integrative analysis of microbes and metabolites was conducted. Co-inertia analysis was utilized to demonstrate a substantial and significant correlation between bacterial and fungal communities, on one hand, and the metabolite profiles derived from both the rhizosphere soil (Figures 9a,b) of *P. tabuliformis*.

Mantel correlation analyses were conducted to examine how shifts in microbial communities correspond to changes in key differential metabolites in the rhizosphere soil of *P. tabuliformis* under different infestation levels. In the bacterial network, only a few weak correlations were detected at RT0, primarily associated with basic carbohydrates such as sedoheptulose, sucrose, trehalose, and D-glucose. At RT1 and RT2, correlation density increased markedly, with enriched associations involving amino acids (e.g., L-phenylalanine, L-proline, L-tyrosine, L-glutamine) and lipid-derived metabolites such as arachidonic acid and eicosapentaenoic acid. These compounds are closely linked to host defense activation. At RT3, the bacterial network reached its highest complexity, featuring numerous strong correlations (Mantel’s r ≥ 0.6) involving prostaglandins (e.g., prostaglandin A2, F3alpha, J2), antimicrobial-related metabolites (e.g., nocardicin A, Geldanamycin, mitomycin), and multiple vitamins or cofactors (e.g., pantothenic acid, pyridoxine, tetrahydrofolic acid) (Figure 9c). The fungal network exhibited a similar but more pronounced trend. At RT0, correlations were weak and concentrated in primary carbohydrate metabolism. Compared with RT1, both the number and strength of correlations increased substantially at RT2, with enriched associations involving amino acids and fatty acid–derived metabolites such as arachidonic acid and eicosapentaenoic acid. At RT3, the fungal network displayed the greatest complexity, characterized by numerous strong correlations (Mantel’s r ≥ 0.6) with prostaglandins, antimicrobial metabolites (e.g., nocardicin A, Geldanamycin), and vitamin-related compounds (Figure 9d).

**Figure 10:**
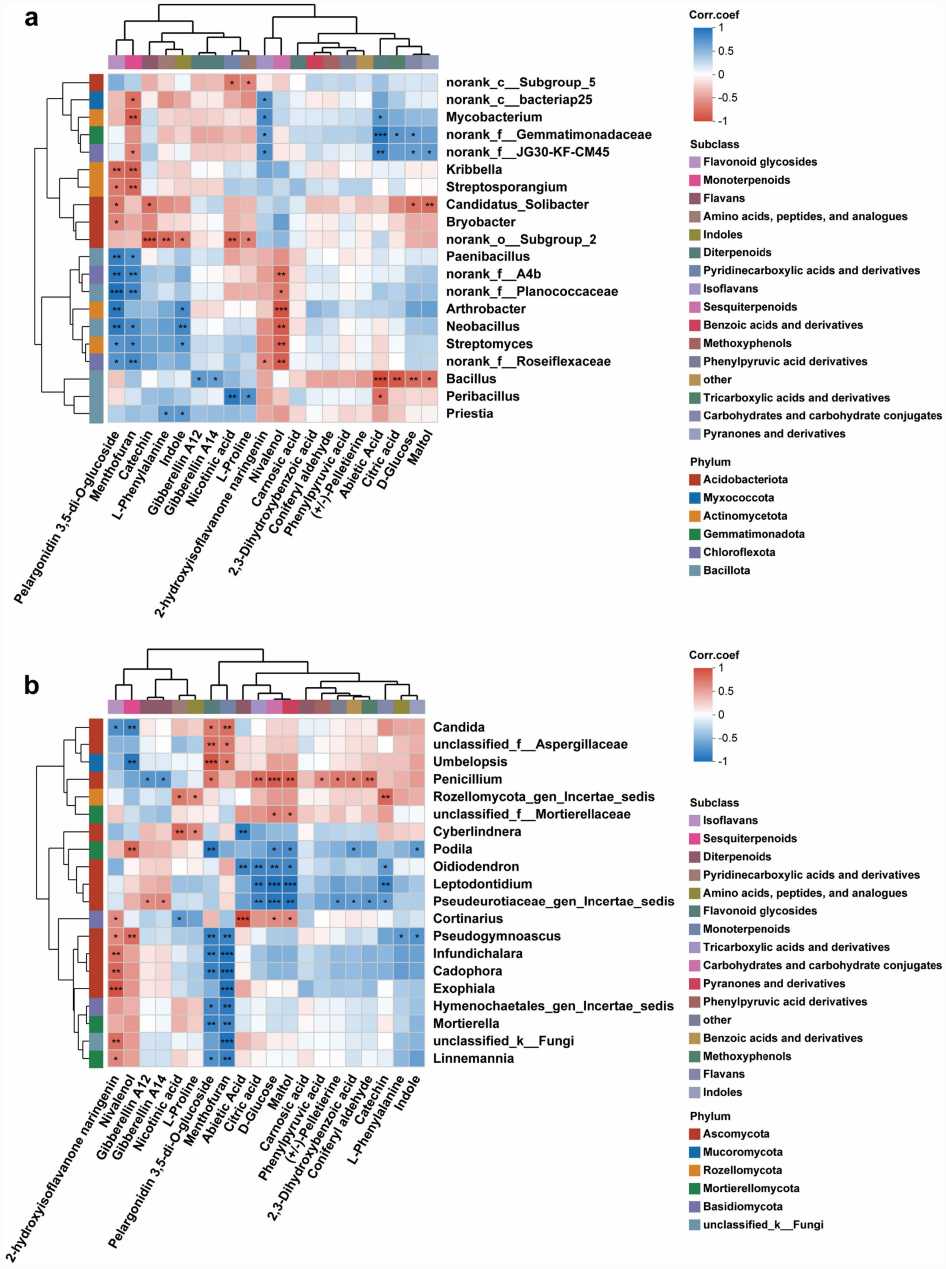
Correlation analysis between differential microorganisms and key metabolites associated with major metabolic pathways in the rhizosphere soil of *Pinus tabuliformis* under different infestation levels. Spearman correlation heatmaps depicting the associations between differential bacterial **(a)** and fungal **(b)** taxa and key metabolites enriched in major metabolic pathways. Red and blue colors denote positive and negative correlations, respectively, with asterisks indicating levels of statistical significance (*p* < 0.05, *p* < 0.01, *p* < 0.001). Metabolites are grouped by subclass, and bacterial taxa are annotated by phylum. RT0 denotes the rhizosphere soil of healthy *Pinus tabuliformis*, RT1–RT3 denotes rhizosphere soil of *Pinus tabuliformis* at different levels of infestation.

Correlation analysis revealed clear associations between differential bacterial taxa and key secondary metabolites (Figure 10a). Several Actinomycetota- and Gemmatimonadota-affiliated taxa, such as *Mycobacterium*, members of Gemmatiomonadaceae, and *Candidatus Solibacter*, showed significant negative correlations with flavonoid glycosides, monoterpenoids, and indole derivatives. In contrast, Bacillota genera including *Paenibacillus*, *Bacillus*, *Peribacillus*, and *Priestia* exhibited strong positive correlations with diterpenoids, sesquiterpenoids, and pyridinecarboxylic acid derivatives (e.g., abietic acid, camphoric acid). The fungal network displayed a complementary pattern with more pronounced metabolite specificity (Figure 10b). Yeast genera such as *Candida*, *Ogataea*, and *Cyberlindnera* were positively correlated with several upstream flavonoids and indole-related compounds. Conversely, filamentous fungi including *Cladosporium*, *Exophiala*, *Penicillium*, and Mortierellomycota- and Dothideales-related taxa showed consistent negative correlations with diterpenoids, sesquiterpenoids, and TCA-related metabolites.

## 4 Discussion

The interactions between plants and soil are closely related to the microbiome ecology in which they are situated (Hu et al., 2018; Xu et al., 2025). Previous studies have demonstrated that the complex changing interactions between aboveground plant parts and their root-associated soil collectively form the microbiome ecology of the plant, which mirrors and influences its growth status (Borgström et al., 2017; Hannula et al., 2019; Hou et al., 2021; Xiong et al., 2021). In this study, the effects of *Dendroctonus valens* infestation intensity on the bacterial and fungal communities, and the associated metabolome, in both the phloem and rhizosphere of *Pinus tabuliformis* were investigated. Infestation level dictated distinct shifts in microbial diversity and composition, with extensive microbe–microbe interactions. Correlation analyses revealed pervasive microbe–metabolite links that were predominantly bacteria- or fungi-mediated. Collectively, our results identify key microbial taxa and metabolites that may govern *D. valens* invasion and emphasize the key role of plant-related microorganisms and metabolites in the infestation process of *D. valens*.

### 4.1 Dynamics of microbial communities in *Pinus tabuliformis*-associated under different infestation levels of *D. valens*

Although studies of microbiomes in *P. tabuliformis* have primarily focused on microbiomes (Wang et al., 2024; Xu et al., 2025), information on *P. tabuliformis* microbiome in phloem and rhizosphere soil and the role of microbiome in preventing the invasion of *D. valens* remains limited. In this study, the different infestation levels significantly affect the microbiome in phloem and rhizosphere soil of *P. tabuliformis*. The invasion of *D. valens* led to the increase of bacterial community diversity in the infested parts of *P. tabuliformis*, while the diversity of fungal community decreased significantly, and the community composition also changed significantly. This change may be due to the invasion of *D. valens*, which caused the change of microbial population balance in phloem of *P. tabuliformis*. This may indicate that there is a close relationship between the balance of microbial community. When the plant is harmed by insects, its physiological state and immune system will change, which may lead to changes in microbial community and plant health (Paasch and He, 2021). As the infestation level of *P. tabuliformis* increased, the abundance of *Erwinia* continuously rose, while the abundances of *Rouxiella* first increased and then decreased. The abundances of *Frateuria* and *Pseudoxanthomonas* significantly increased after healthy *P. tabuliformis* was infested. *Frateuria* can produce terpenoids, which may promote the aggregation behavior of *D*. *valen* (Joyeux et al., 2004). The enzyme of pinopinic alcohol metabolism mainly comes from *Erwinia*, and *Pseudoxanthomonas* provides the key enzyme in fatty acid metabolism, which is very important for the invasion of *D. valens* (Cheng et al., 2025). Under different infestation levels of *D. valens*, the microbial community structure and composition in the rhizosphere soil of *P. tabuliformis* showed a trend of first decreasing and then increasing, which indicated that the health of aboveground insects could change the underground microbial flora (Gao et al., 2024; Pec et al., 2017; Wu et al., 2024). Upon insect infestation, plants can elicit defensive responses, often facilitated by specific microorganisms. In certain instances, they further bolster their resistance by enlisting the aid of rhizosphere soil-associated microorganisms (Gao et al., 2021). Insect invasions can induce changes in the community structure of plant-associated microorganisms. Plant microbiotas are intricately linked to soil environments. Plant growth can alter the physicochemical properties of soil, thereby influencing the composition of soil microbial communities. There exists a dynamic interplay among insects, plants, and soil (Choi et al., 2021; Hassani et al., 2018; Mahmud et al., 2021). Some microorganisms can enhance the defense ability of plants by manipulating certain channels and even enhance the defense ability of plants by influencing the rhizosphere soil microorganisms, thus preventing the invasion of insects. In the bacterial community, with the increase of the infestation level of *P. tabuliformis*, the abundance of *Bradyrhizobium* first increased and then decreased, while that of *Pseudomonas* first decreased and then increased. *Pseudomonas* and *Bradyrhizobium* play a positive role in plant defense and nutrient uptake at the root level. They promote plant growth in the rhizosphere, enhance the acquisition of nutrients, and form symbiotic relationships with plants, thereby strengthening plant growth. These bacteria improve plant health and development by secreting various metabolites and hormones, increasing the availability of nutrients, and enhancing plant defense responses (Banasiewicz et al., 2025; Sultana et al., 2024). Specifically, *Bradyrhizobium* may initially respond positively to the altered root environment induced by initial infestation levels but may be outcompeted or negatively affected as infestation severity escalates. Conversely, *Pseudomonas* may become more influential as the infestation progresses, potentially due to its ability to adapt to or mitigate the effects of increased stress.

Microbiomes frequently exhibit a diverse array of interactions and play pivotal roles in various ecosystems (Li et al., 2020; Li et al., 2022; Ugwu et al., 2020). Our findings demonstrate extensive correlations between bacterial or fungal populations. Comparable results have been reported in studies on the microbiome of plant and soil, revealing robust associations among different microbial components. Given limited living space and nutritional resources, diverse microorganisms, including endosymbionts and symbiotic microorganisms, exist in direct or indirect relationships (Chen et al., 2023; Li et al., 2020; Manirajan et al., 2018; Partida-Martinez et al., 2007; Philippot et al., 2024; Shen et al., 2024). Specifically, microorganisms can participate in resisting biotic and abiotic stresses and protect plants from adverse factors in the process of health to plants (Singh et al., 2025). We propose that the diverse microorganisms in the gut and living environment of *D. valens* are associated through complex regulatory mechanisms, crucial for its plant invasion.

### 4.2 Differential responses of metabolites in the phloem and rhizosphere soil of *Pinus tabuliformis* under different infestation levels

Upon insect infestation, the infested portions of plants exhibit both physical and chemical defenses, releasing metabolites that are distinctly different from those of non-infested plants (Sun et al., 2023). Additionally, the root exudates also undergo alterations (Friman et al., 2021). In this study, it was found that when *D. valens* infested *P. tabuliformis* at different levels, significant metabolic responses occurred in the phloem and rhizosphere soil. KEGG pathway classification exists in the metabolism of terpenoids and polyketides, secondary metabolites, amino acids and lipids. These metabolic changes coincide with shifts in the microbial community within the phloem and rhizosphere soil *P. tabuliformis*. Similarly, when *Plutella xylostella* feeds on its plants, metabolite differences are also observed (Yuan et al., 2023). Furthermore, one of the primary plant responses to stress is the regulation of rhizosphere soil secretions, the exudates released into the rhizospheric environment play a pivotal role in the establishment and formation of plant-microbe interactions (Afridi et al., 2024). The synthesis and metabolism of some carbohydrates can affect the palatability of plants, and the synthesis and metabolism of some lipids and peptides can enhance the defense of plants (Apone et al., 2009; Geissman, 1958; Vicente et al., 2022; Wang et al., 2018; Zi et al., 2022).

By comparing the differential metabolites identified in different infestation severity groups with those in healthy groups of *P. tabuliformis* phloem and rhizosphere soil, KEGG enrichment pathway analysis was conducted. It was found that under different infestation levels of *D. valens*, there were significant differences in the enrichment of metabolites in the phloem of *P. tabuliformis*. The phloem played a more important role in the biosynthesis pathways of phenylpropanoids and flavonoids. In contrast, the enrichment results of metabolites in the rhizosphere soil of oil pine were similar, with a more significant role in the biosynthesis pathways of steroid hormones. Phenylpropanoids and flavonoids play crucial roles in plant defense by deterring insects from feeding on plant tissues (Shi et al., 2025). Phenylpropionic acid pathway is an important way for plants to synthesize a variety of secondary metabolites, including lignin, flavonoids, phenolic acids and so on. These secondary metabolites play an important role in plant growth and development, defense mechanism and adaptation to environmental changes (Vogt, 2010; Zhang et al., 2025). In this study, it was found that some substances in phenylpropionic acid pathway of *P. tabuliformis* changed significantly under different damage degrees of *D. valens*. For example, luteolin and taxifolin increased significantly with the increase of infestation degree of *P. tabuliformis*, and after *P. tabuliformis* was infested, cinnamaldehyde, 4-coumaroylshikimate and 5-hydroxyferulic acid decreased significantly with the increase of infestation level. Certain flavonoids can inhibit the digestive enzymes of insects, reducing their ability to extract nutrients from plants. Moreover, these compounds are associated with plant hormone signaling pathways such as jasmonic acid (JA) and salicylic acid (SA), which are vital for regulating plant defense responses against herbivorous insects(Shi et al., 2025; Zhang et al., 2025). The biosynthesis of steroid hormones by soil microorganisms may alter the chemical characteristics of plants, making them more attractive or repellent to insects (Bouvaine et al., 2014). It can also influence the community structure and function of microorganisms within insects, thereby affecting their physiological metabolism and immune responses (Lafont and Dinan, 2024). In addition, the metabolic pathways identified in the rhizosphere, specifically metabolism of terpenoids and polyketides and biosynthesis of other secondary metabolites, are likely to play significant roles in the defense of *P. tabuliformis* against the invasion of *D. valens*. Research has indicated that nivalenol exhibits toxic effects on insects (Boguś et al., 2021), and (S)-(−)-perillyl alcohol can influence insect behavior and physiology (Zhao et al., 2024), all these compounds demonstrate pesticidal properties. Caffeine can enhance plant defensive capabilities, serving both as a direct toxin and as an activator of the plant’s defense system (Sugiyama et al., 2016). Ferulic acid has shown insecticidal effects by disrupting insect feeding behavior (Zhang et al., 2020). Coumarin plays multiple roles in plant defense against insect herbivory and fungi, exhibiting broad-spectrum antimicrobial activity against both fungi and bacteria (Zaynab et al., 2024). Protocatechuic aldehyde, a compound with antioxidant properties, can enhance plant stress resistance (Zhang et al., 2021). In summary, the chemical defense of plants after insects infest the plants is not only manifested in the infested parts, but also extends to their roots (Erb and Reymond, 2019).

### 4.3 The microorganism–metabolite correlations potentially in phloem and rhizosphere soil of *Pinus tabuliformis* involved in mechanisms under different infestation levels

Our integrative multi-omics analyses demonstrate that both the phloem and rhizosphere microbiomes of *Pinus tabuliformis* exhibit tightly coordinated responses with host metabolic reprogramming during *Dendroctonus valens* infestation. Co-inertia analyses revealed strong correlations between microbial community restructuring and metabolite shifts in both niches, supporting the concept that plant secondary metabolites act simultaneously as selective pressures and functional substrates for associated microbes, forming metabolite-mediated feedback loops (Jacoby et al., 2021; Pang et al., 2021).

Mantel correlation networks further clarified this coordination. Under low infestation (PT0/RT0), microbial–metabolite associations were weak and limited to primary carbohydrates, reflecting baseline metabolic coupling. Under mildly infested (PT1–PT2) and moderately infested (RT1–RT2), both bacterial and fungal taxa showed enriched correlations with amino acids and fatty-acid-derived defense molecules—such as arachidonic and eicosapentaenoic acids—that play central roles in plant stress signaling and microbial recruitment (Beccaccioli et al., 2022; Sharifi et al., 2018). Under severe infestation (PT3/RT3), network complexity peaked, with strong correlations linking microbial taxa to prostaglandins, antimicrobial compounds and vitamin/cofactor metabolites. These metabolites typically accumulate during late-stage biotic stress and strongly modulate microbial assembly, favoring taxa tolerant of or capable of transforming defensive compounds (Berendsen et al., 2012).

Taxon-specific patterns further reveal functional differentiation within the microbiomes. In the phloem, Burkholderiales and *Paenibacillus* correlated positively with early phenylpropanoid intermediates, while Actinomycetota taxa (e.g., *Mycobacterium*) were negatively linked to downstream products, suggesting differential roles in phenylpropanoid metabolism. Similarly, rhizosphere Bacillota taxa showed positive associations with diterpenoids and sesquiterpenoids, whereas Actinomycetota and Gemmatimonadota taxa were suppressed by flavonoids and indole derivatives. Fungal communities exhibited parallel trends: yeasts correlated with upstream metabolites, while filamentous fungi showed negative associations with diterpenoids and TCA-related compounds—consistent with the known sensitivity of saprotrophic fungi to conifer defensive terpenoids (Raffa and Smalley, 1995; Six and Wingfield, 2011). Together, these findings support a model in which *P. tabuliformis* under beetle infestation undergoes a staged transition from basal microbial–metabolite coupling to defense-driven, and ultimately microbially modulated, metabolic restructuring. This highlights both the phloem and rhizosphere microbiomes as potential leverage points for developing microbiome-informed *D. valens* management strategies.

## 5 Conclusion

In this study, *Pinus tabuliformis* exhibited pronounced shifts in both phloem and rhizosphere microbial communities, along with distinct metabolomic changes, in response to varying levels of *Dendroctonus valens* infestation. Different levels of *D. valens* infestation triggered shifts in microbial diversity, altered the relative abundance of key bacterial and fungal taxa, and activated compartment-specific defense-related metabolic pathways. Moreover, specific bacterial and fungal taxa exhibited directional correlations with phenylpropanoid intermediates, flavonoids, diterpenoids, and indole derivatives, revealing functional specificity in how microbiota interact with host defense metabolism in the phloem and rhizosphere. These findings underscore the pivotal role of microbiome–metabolite interactions in shaping plant responses to bark beetle infestation and provide new insights into the microbial mechanisms influencing host susceptibility and pest progression. Collectively, this work expands our understanding of plant–soil–microbe dynamics under insect stress and offers a foundation for developing microbiome-informed strategies for the sustainable management of *D. valens*.

## Data Availability

The data that support the findings of this study are freely accessible in NCBI SRA at https://www.ncbi.nlm.nih.gov/sra/. All raw sequence reads are deposited in the NCBI under the BioProject number BioProject PRJNA1257560 (16S rRNA and ITS sequencing).
