# Supplementary figures and images for: Microbiome and metabolome dynamics in phloem and rhizosphere of Pinus tabuliformis against Dendroctonus valens infestation
Source: Front Microbiol. 2026 Mar 4;17:1754801. doi: 10.3389/fmicb.2026.1754801 (PMC12997781; doi:10.3389/fmicb.2026.1754801)

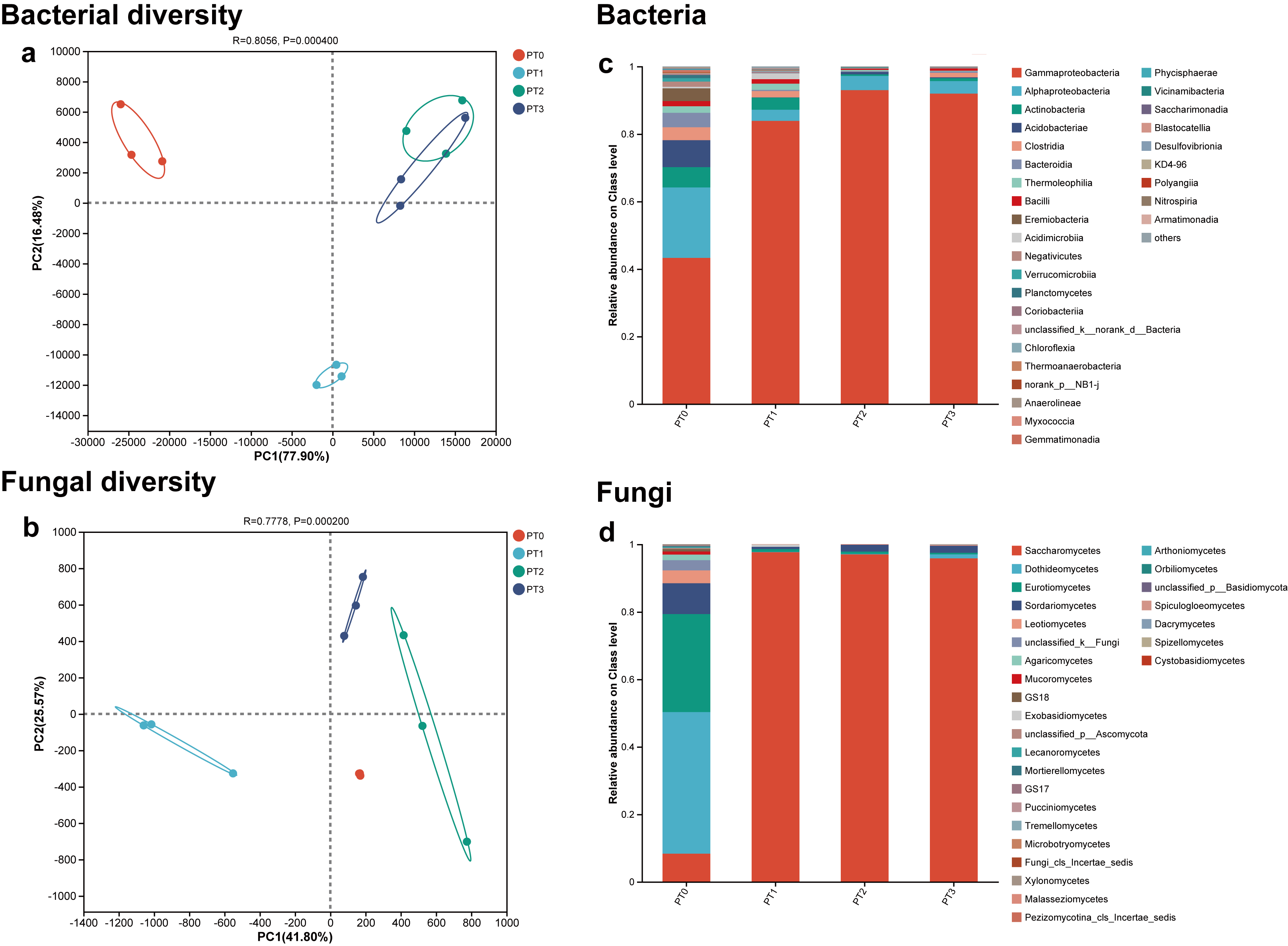

Supplement: Supplementary file 1 [file Data_Sheet_1.zip › Supplementary Material/Figure S1 Principal co-ordinates analysis (PCoA) visualizing amplicon sequence variant-based bacterial (a) and fungal (b) clusters. Circles in different colors represent different samples..tif]

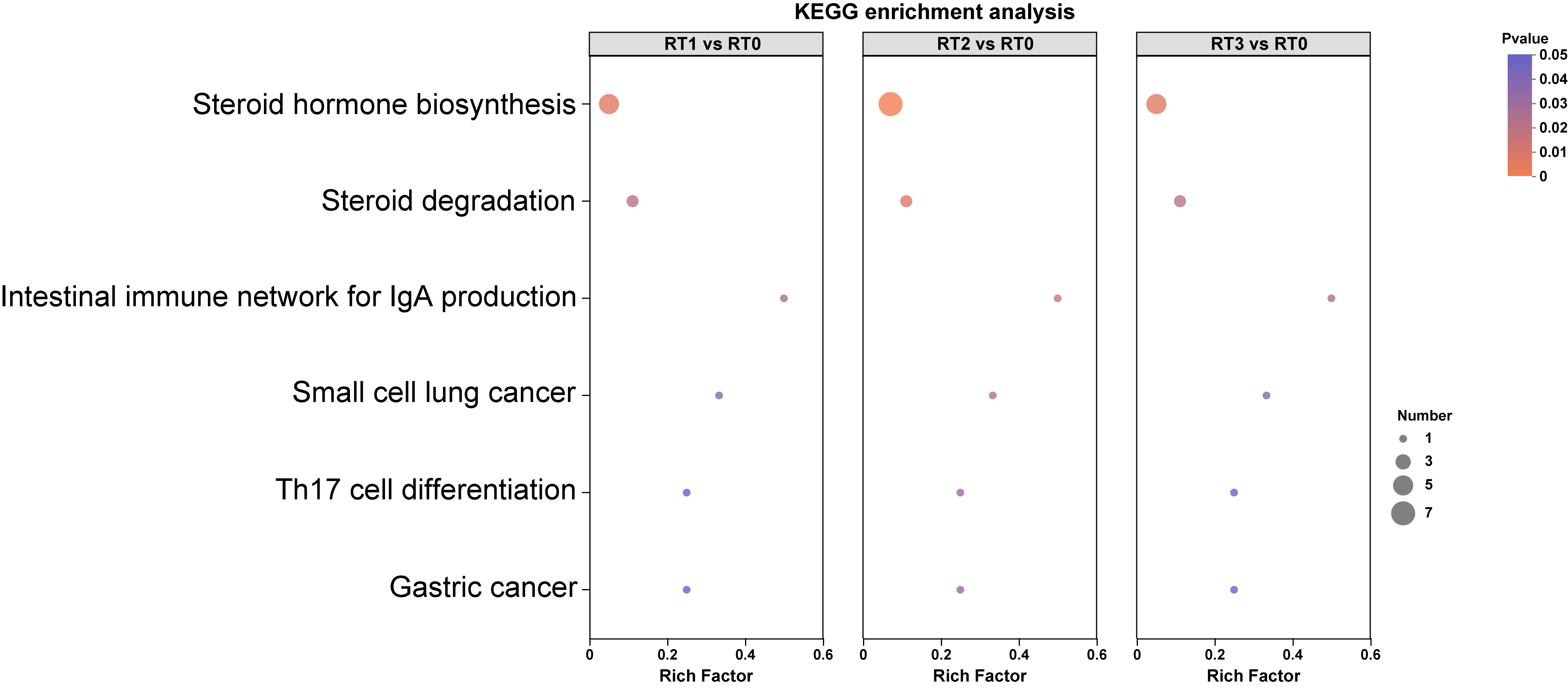

Supplement: Supplementary file 1 [file Data_Sheet_1.zip › Supplementary Material/Figure S10 Functional analysis for differential metabolites of the rhizosphere soil of P. tabulaeformis under different levels of infestation.tif]

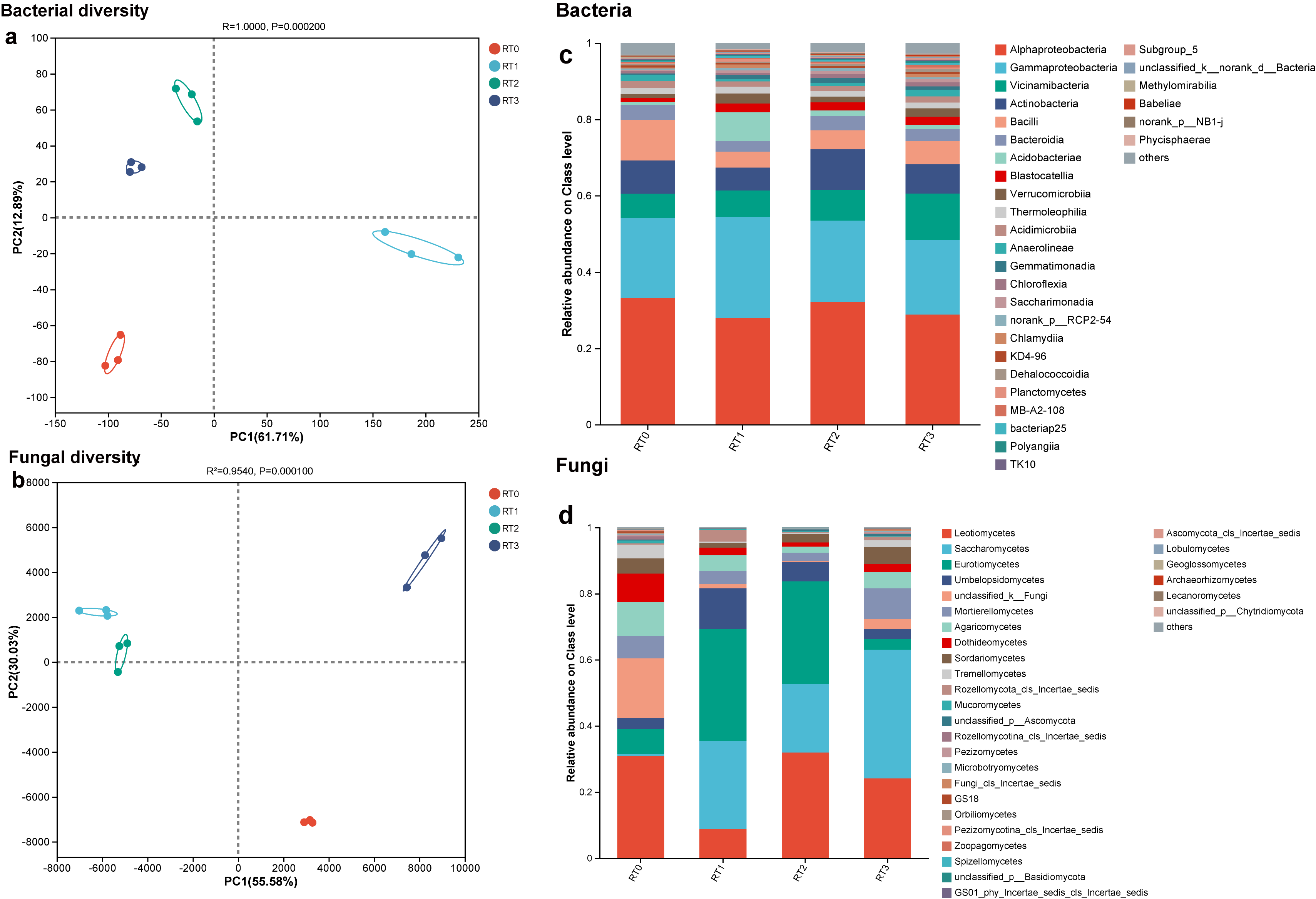

Supplement: Supplementary file 1 [file Data_Sheet_1.zip › Supplementary Material/Figure S2 Principal co-ordinates analysis (PCoA) visualizing amplicon sequence variant-based bacterial (a) and fungal (b) clusters. Circles in different colors represent different samples..tif]

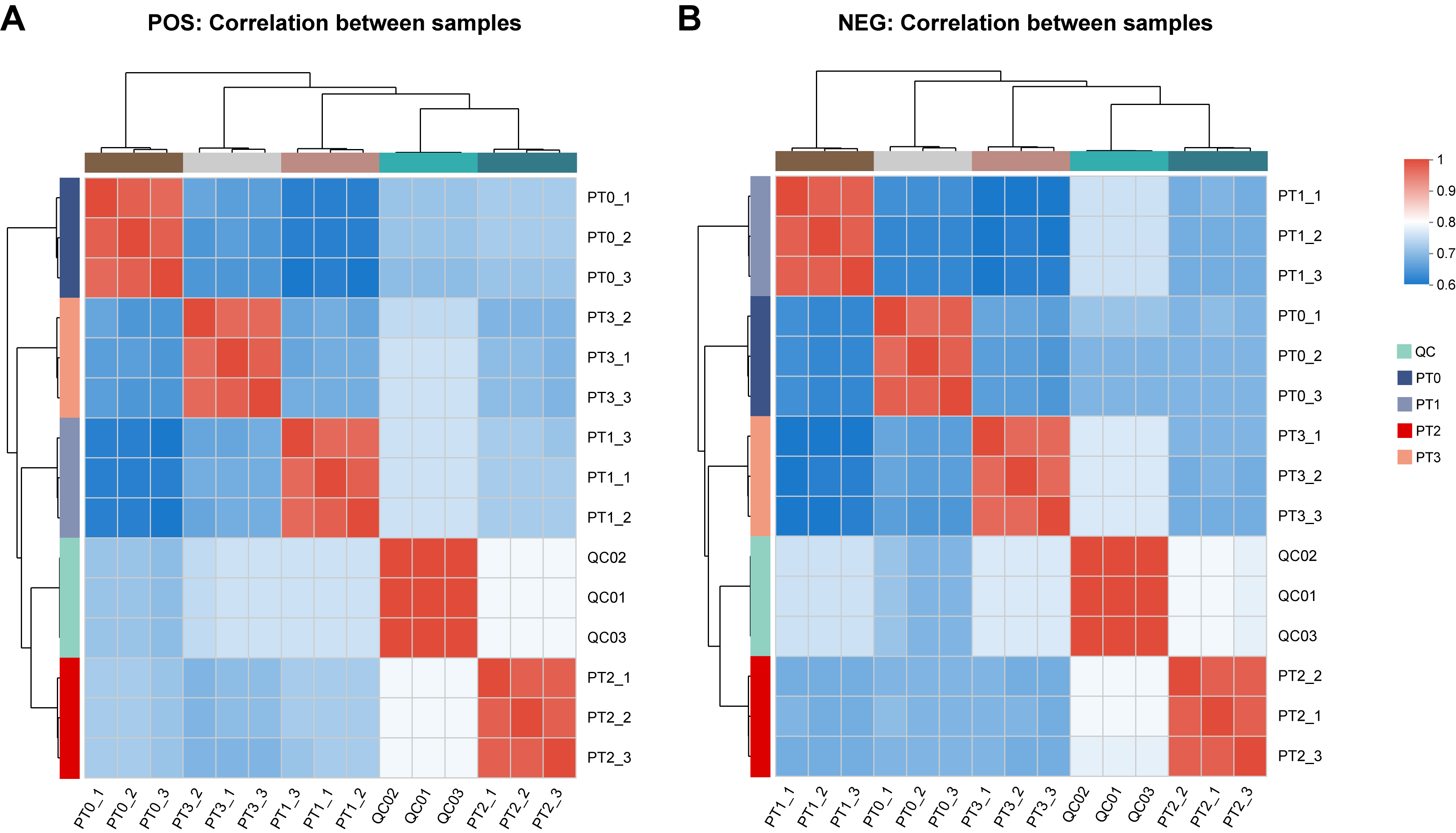

Supplement: Supplementary file 1 [file Data_Sheet_1.zip › Supplementary Material/Figure S3 The correlation levels among metabolome samples in the phloem of P. tabulaeformis based on Pearson's correlation coefficient..tif]

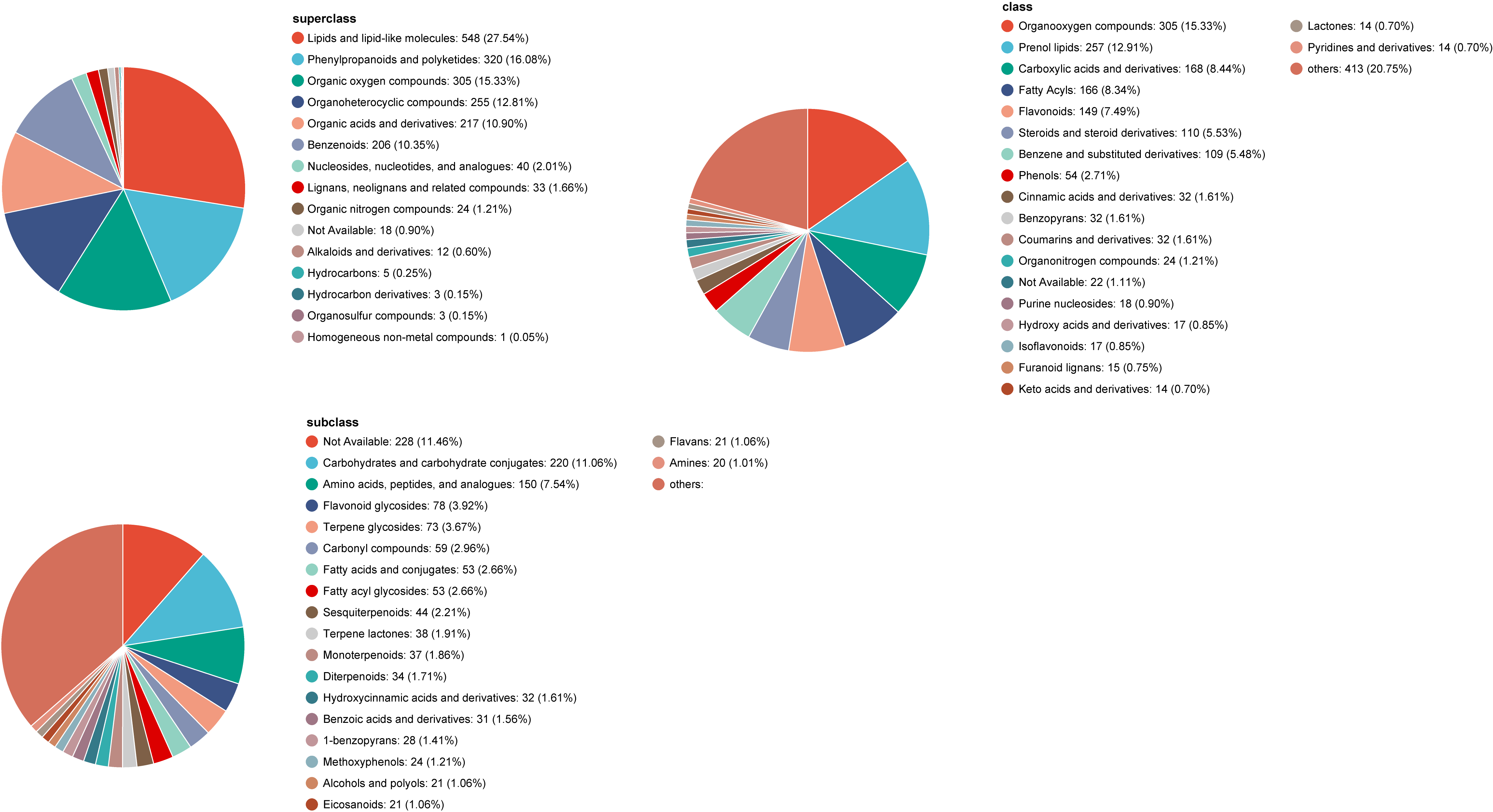

Supplement: Supplementary file 1 [file Data_Sheet_1.zip › Supplementary Material/Figure S4 Taxonomic information of metabolites in the phloem of P. tabulaeformis under different infestation levels..tif]

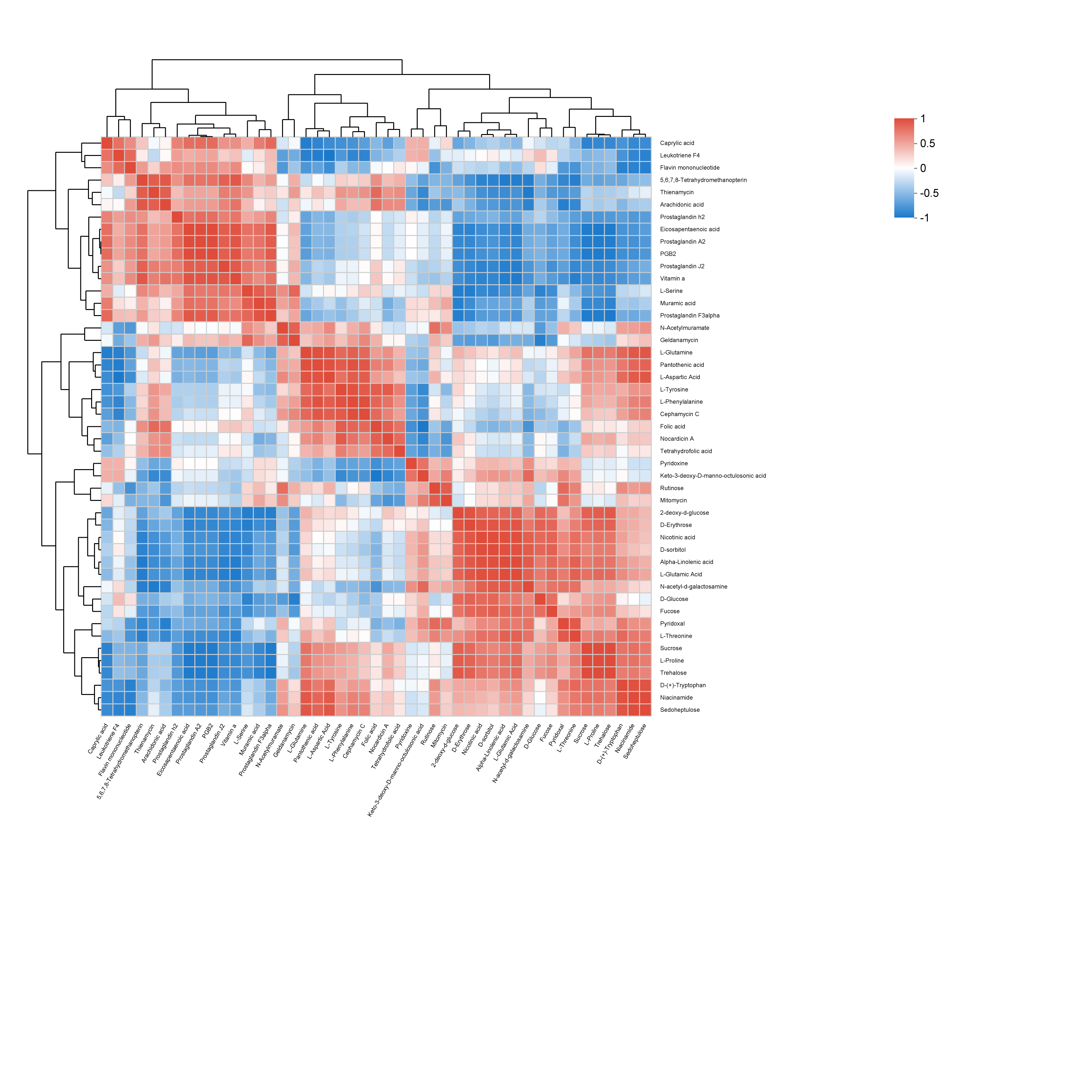

Supplement: Supplementary file 1 [file Data_Sheet_1.zip › Supplementary Material/Figure S5 Correlation of differential metabolites in the phloem of P. tabulaeformis under different infestation levels..tif]

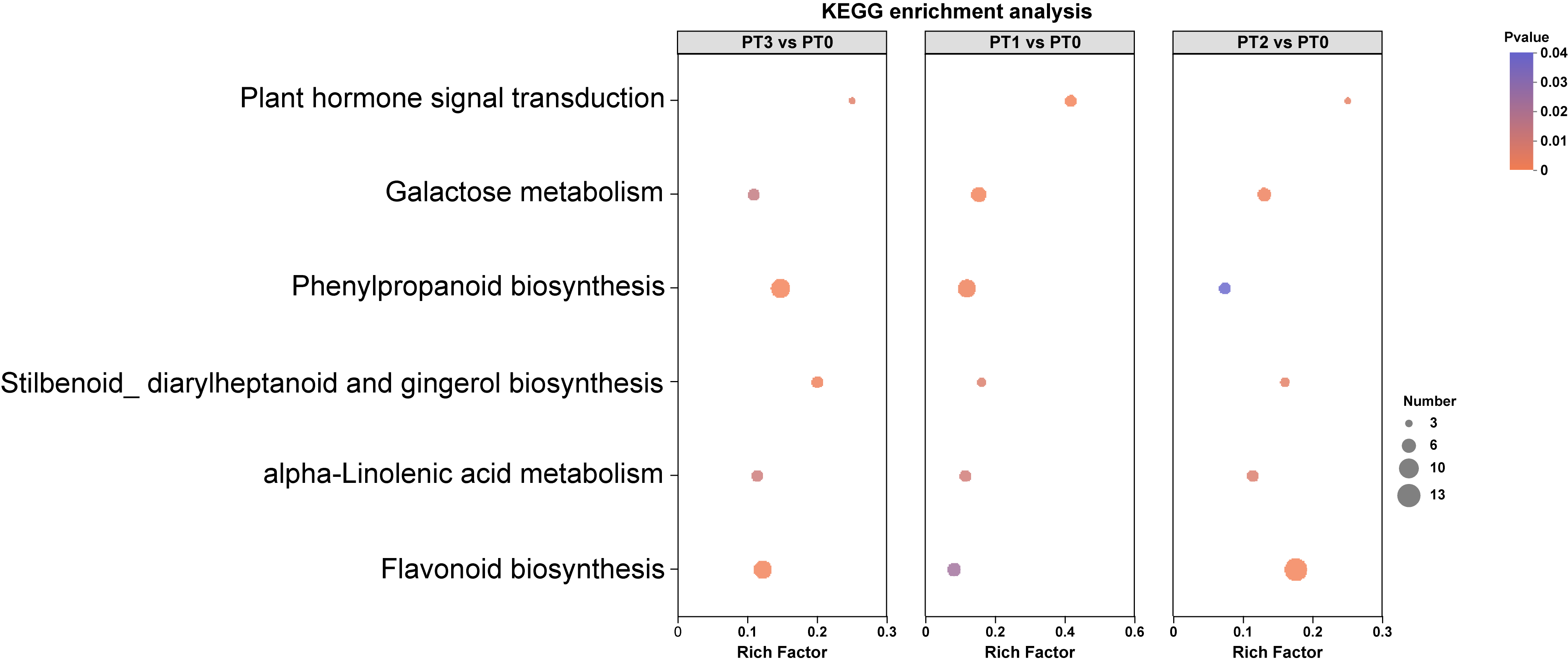

Supplement: Supplementary file 1 [file Data_Sheet_1.zip › Supplementary Material/Figure S6 Functional analysis for differential metabolites of the phloem of P. tabulaeformis under different levels of infestation..tif]

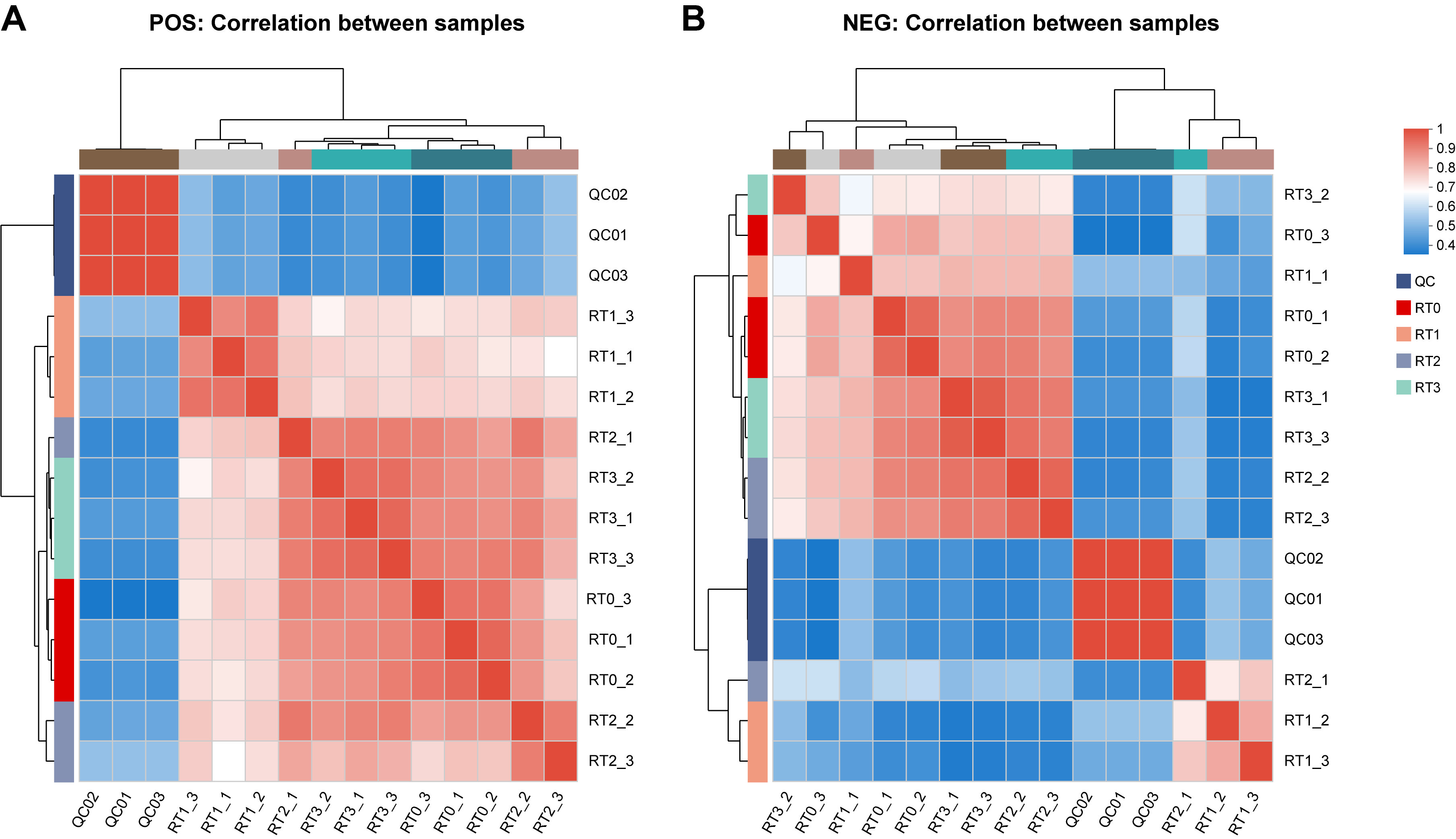

Supplement: Supplementary file 1 [file Data_Sheet_1.zip › Supplementary Material/Figure S7 The correlation levels among metabolome samples in the rhizosphere soil of P. tabulaeformis based on Pearson's correlation coefficient..tif]

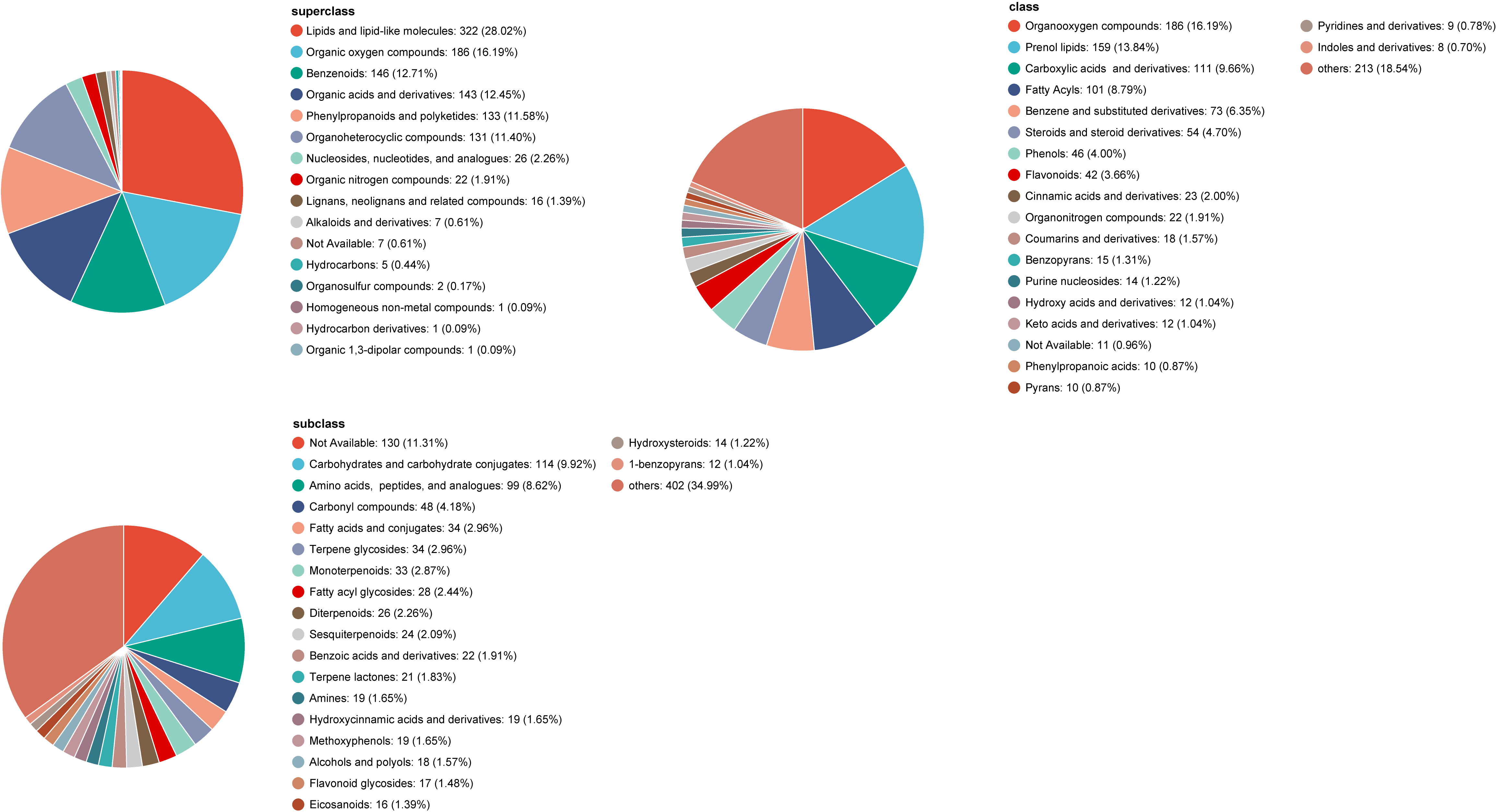

Supplement: Supplementary file 1 [file Data_Sheet_1.zip › Supplementary Material/Figure S8 Taxonomic information of metabolites in the rhizosphere soil of P. tabulaeformis under different infestation levels.tif]

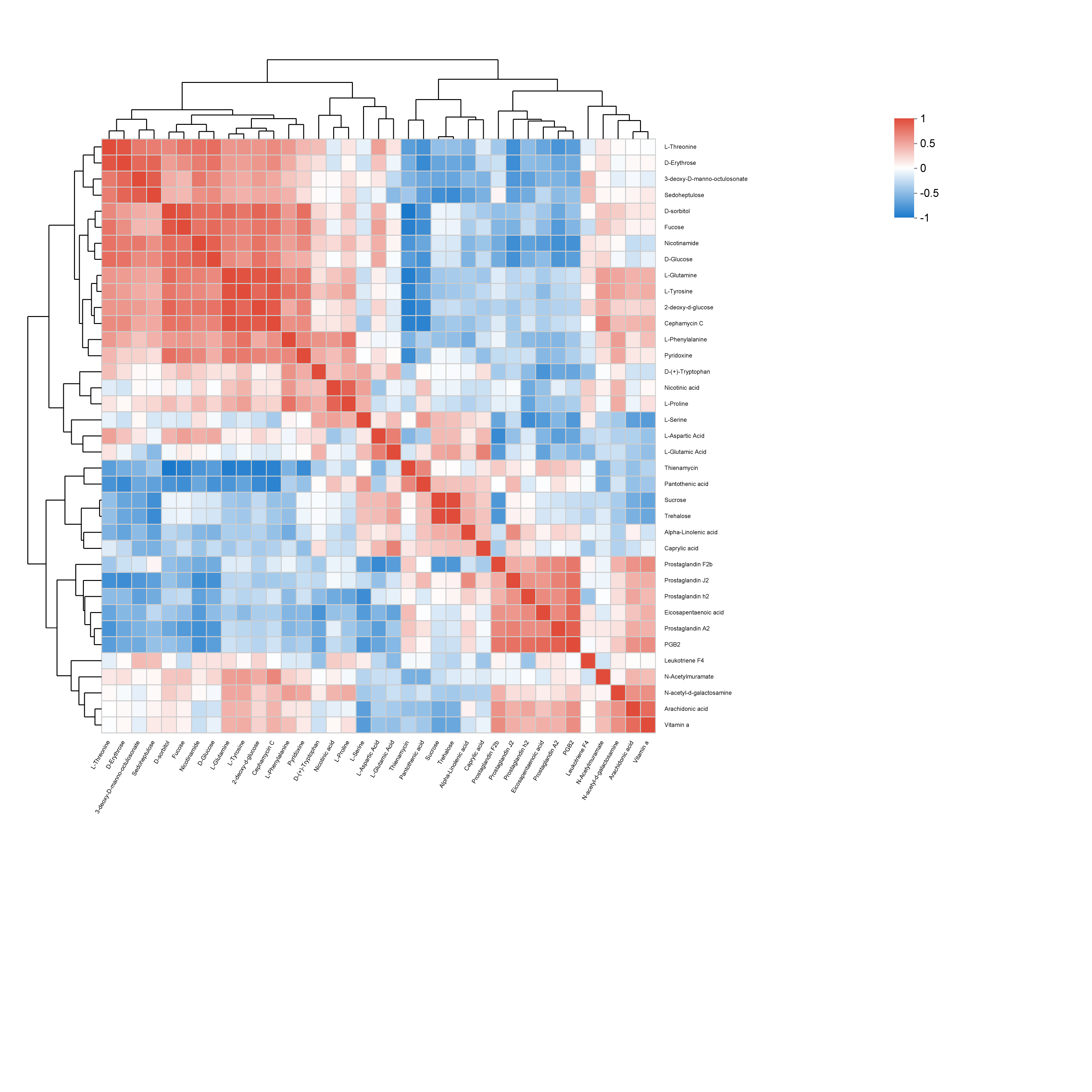

Supplement: Supplementary file 1 [file Data_Sheet_1.zip › Supplementary Material/Figure S9 Correlation of differential metabolites in the rhizosphere soil of P. tabulaeformis under different infestation levels..tif]
